# Supplementary material for: Genetic and functional diversity of β-N-acetylgalactosamine-targeting glycosidases expanded by deep-sea metagenome analysis
Source: Nat Commun. 2024 May 10;15:3543. doi: 10.1038/s41467-024-47653-2 (PMC11087588; doi:10.1038/s41467-024-47653-2)
Supplement: Supplementary file 1 — Supplementary Information [file 41467_2024_47653_MOESM1_ESM.pdf]

Supplementary Information

Genetic and functional diversity of β-*N*-acetylgalactosamine-targeting glycosidases expanded by deep-sea metagenome analysis

Tomomi Sumida[1*], Satoshi Hiraoka[1], Keiko Usui[1], Akihiro Ishiwata[2], Toru Sengoku[3], Keith A. Stubbs[4], Katsunori Tanaka[2,5], Shigeru Deguchi[1], Shinya Fushinobu[6*], Takuro Nunoura[1].

[1] Research Center for Bioscience and Nanoscience, Research Institute for Marine Resources Utilization, Japan Agency for Marine-Earth Science and Technology (JAMSTEC), Yokosuka, Japan

[2] RIKEN, Cluster for Pioneering Research, Wako, Saitama, Japan.

[3] Department of Biochemistry, Yokohama City University Graduate School of Medicine, Kanazawa-ku, Yokohama, Japan

[4] School of Molecular Sciences, The University of Western Australia, Crawley, WA, Australia

[5] Department of Chemical Science and Engineering, Tokyo Institute of Technology, Meguro-ku, Tokyo, Japan

[6] Graduate School of Agricultural and Life Sciences, The University of Tokyo, Bunkyo-ku, Tokyo, Japan

***Corresponding authors:**

Tomomi Sumida, sumidat@jamstec.go.jp
Shinya Fushinobu, asfushi@mail.ecc.u-tokyo.ac.jp

Supplementary Figures

Supplementary Figure 1. Symbol nomenclature for the GalNAc-containing glycans

Supplementary Figure 2. Candidate β-NGA sequences retrieved from deep-sea sediment metagenomes

Supplementary Figure 3. Candidate β-NGA sequences containing DUF4091

Supplementary Figure 4. Comparison between NgaAt and HEXO1–3

Supplementary Figure 5. s-v plots of recombinant β-NGAs.

Supplementary Figure 6. Hydrolysis of oligosaccharides by recombinant β-NGAs

Supplementary Figure 7. Hydrolysis of chondroitin sulfate A, B, C by recombinant β-NGAs

Supplementary Figure 8. Secondary structure and characteristic domains of NgaCa (Group 1)

Supplementary Figure 9. Secondary structure and characteristic domains of NgaAt (Group 2)

Supplementary Figure 10. Secondary structure and characteristic domains of NgaDssm (Group 3)

Supplementary Figure 11. Secondary structure and characteristic domains of NgaP2 (Group 4)

Supplementary Figure 12. Secondary structure and characteristic domains of BvGH123 (GH123)

Supplementary Figure 13. The structures of NgaCa, NgaAt, NgaDssm, and NgaP2

Supplementary Figure 14. Stereo view of the active site of β-NGAs

Supplementary Figure 15. 2D ^{1}H-^{1}H COSY spectra of βGalα/β1-3GalNAc and βGalβ1-3GalNAc-β-*p*NP

Supplementary Figure 16. NMR spectra of Galβ1-3GalNAc-β-*p*NP in D_2O

Supplementary Figure 17. NMR spectra of Galβ1-3GalNAc-β-*p*NP treated with the enzyme at pH in D_2O

Supplementary Figure 18. Neighborhood genes and potential protein interaction networks of β-NGAs

Supplementary Figure 19. Time course for the hydrolysis of GalNAc-β-*p*NP (or Galβ1-3GalNAc-β-*p*NP for NgaCa) by recombinant β-NGAs

Supplementary Tables

Supplementary Table 1. **The structures and the Rf values of the standard oligosaccharides and sugars**

Supplementary Table 2. Data collection and refinement statistics of the crystallography of NgaCa (Group 1)

Supplementary Table 3. Data collection and refinement statistics of the crystallography of NgaAt (Group 2)

Supplementary Table 4. Data collection and refinement statistics of the crystallography of NgaDssm (Group 3)

Supplementary Table 5. Data collection and refinement statistics of the crystallography of NgaP2 (Group 4)

Supplementary Table 6. Data collection and refinement statistics of the crystallography of NgaLy (Group 4)

Supplementary Table 7. Structural similarity between β-NGA groups compared using the root-mean-square distance (RMSD)

Supplementary Table 8. Amino acids conserved between β-NGA groups

Supplementary Table 9. The amounts of enzyme used in the point mutation assay

Supplementary Table 10. ^{1}**H and** ^{13}**C NMR data of Galβ1-3GalNAcα/β**

Supplementary Table 11. ^{1}**H and** ^{13}**C NMR data of Galβ1-3GalNAc-β-*p*NP**

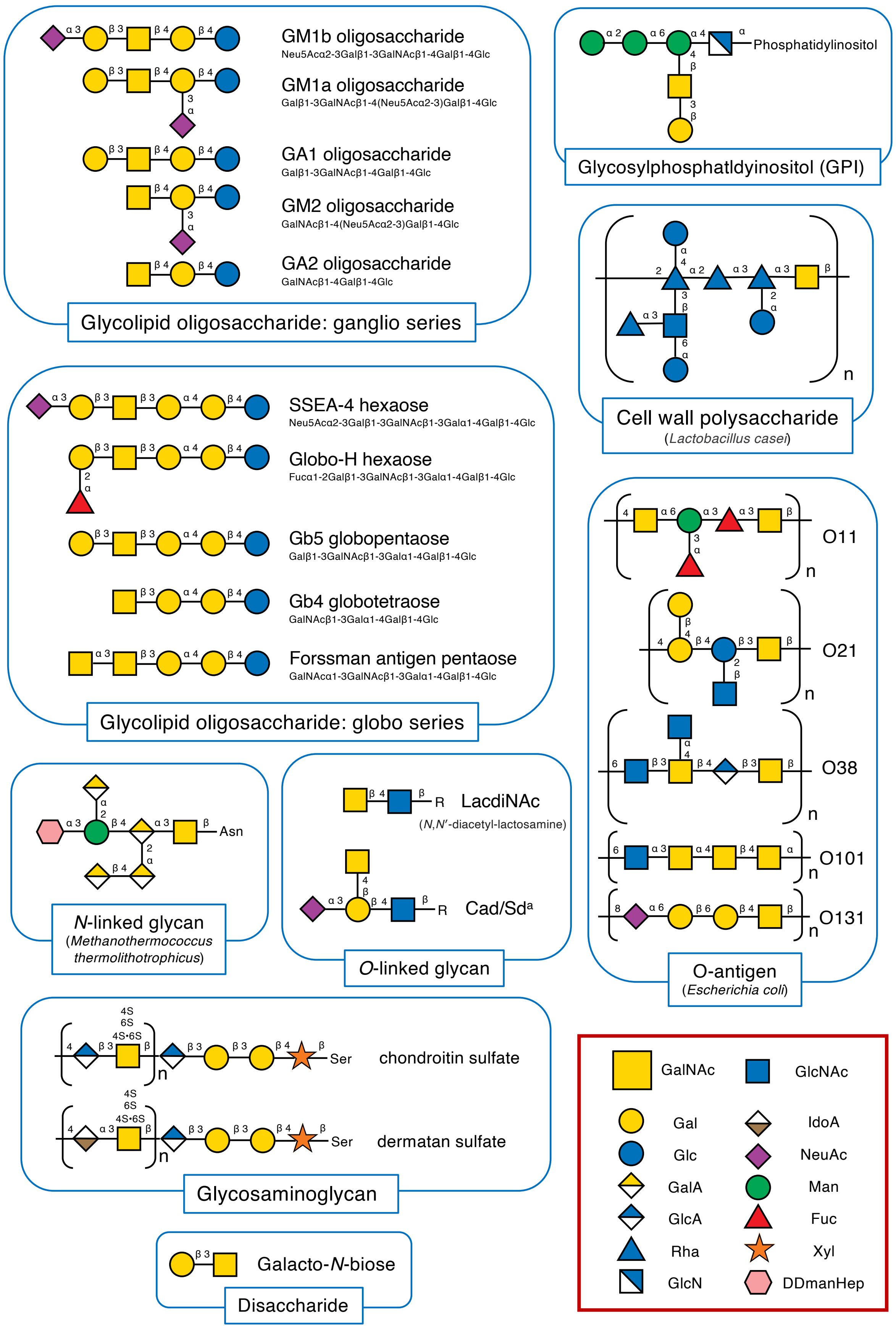

Supplementary Figure 1. Symbol nomenclature for the GalNAc-containing glycans.

a

Sequence Identity (%)

dssm_1	100.0	17.4	16.1	15.4	14.8	17.7
dssm_3	17.4	100.0	24.0	17.5	19.2	21.5
dssm_2	16.1	24.0	100.0	19.3	26.3	20.6
CpNga123	15.4	17.5	19.3	100.0	32.7	35.3
BvGH123	14.8	19.2	26.3	32.7	100.0	35.6
NgaP	17.7	21.5	20.6	35.3	35.6	100.0

b

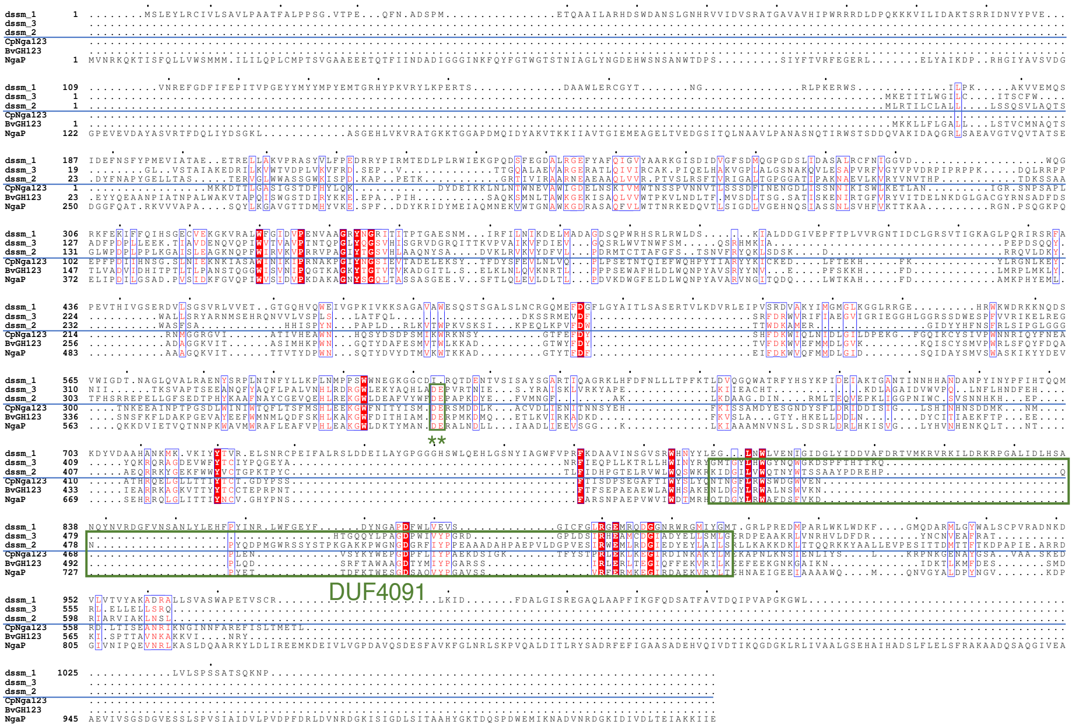

c

Sequence Identity (%) of the DUF4091 area

dssm_3	100.0	35.7	19.6	31.3	35.4
dssm_2	35.7	100.0	27.5	34.4	27.9
CpNga123	19.6	27.5	100.0	42.6	45.9
BvGH123	31.3	34.4	42.6	100.0	52.5
NgaP	35.4	27.9	45.9	52.5	100.0

d

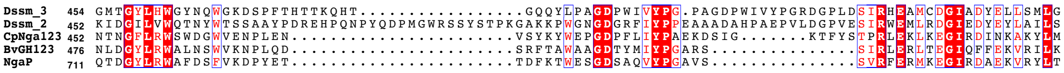

e

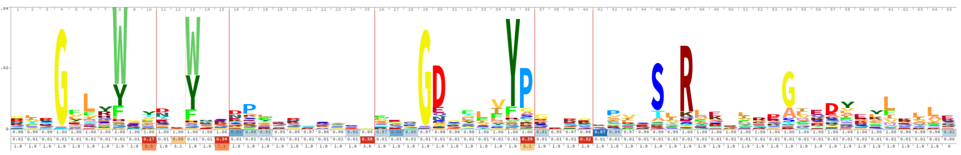

Supplementary Figure 2. Candidate β-NGA sequences retrieved from deep-sea sediment metagenomes.

a, Sequence identities within β-NGA candidates retrieved from deep-sea sediment metagenomes and the known GH123 genes. **b**, Alignment of gene sequence. Residues conserved in all the proteins are shown on a red background. A conserved DE motif (green asterisk, *) is indicated by a green box. The DUF4091 is underlined. **c, d**, Sequence identity of the DUF4091 area. **e**, HMM logo for DUF4091 from the Pfam database site.

a Sequence Identity (%)

GH123	CpNga123	100	33	35	16	19	17	17	18	15	16	16	12	14	13	14	16	18	17
	BvGH123	33	100	36	21	20	19	21	21	17	17	16	17	15	15	16	19	19	21
	NgaP	35	36	100	20	19	21	19	18	18	15	17	16	14	16	16	19	21	21
Group 1	NgaMg	16	21	20	100	39	41	40	40	24	23	24	18	18	18	17	20	19	26
	NgaSc	19	20	19	39	100	48	48	54	20	23	22	15	14	16	15	15	16	22
	NgaCa	17	19	21	41	48	100	55	53	22	23	23	18	19	18	21	18	21	24
	NgaNp	17	21	19	40	48	55	100	56	20	20	20	16	16	15	17	18	18	23
	NgaCs	18	21	18	40	54	53	56	100	22	22	21	17	17	16	18	19	19	25
Group 2	NgaOs	15	17	18	24	20	22	20	22	100	66	66	20	18	19	20	18	21	22
	NgaAt	16	17	15	23	23	23	20	22	66	100	74	19	17	20	21	17	20	23
	NgaGm	16	16	17	24	22	23	20	21	66	74	100	20	18	20	19	18	20	23
Group 4	NgaBl	12	17	16	18	15	18	16	17	20	19	20	100	39	38	42	21	22	23
	NgaBf	14	15	14	18	14	19	16	17	18	17	18	39	100	40	39	20	25	23
	NgaLy	13	15	16	18	16	18	15	16	19	20	20	38	40	100	40	22	24	23
	NgaP2	14	16	16	17	15	21	17	18	20	21	19	42	39	40	100	21	26	26
Group 3	NgaCp	16	19	19	20	15	18	18	19	18	17	18	21	20	22	21	100	36	29
	NgaDssm	18	19	21	19	16	21	18	19	21	20	20	22	25	24	26	36	100	41
	NgaBa	17	21	21	26	22	24	23	25	22	23	23	23	23	23	26	29	41	100

b

```
GH123    CpNga123  1  NTNGFLRWSWDGWVENP...LENVSY.....KYWEPGDPFLIYPAEKDSIGKTFYSTPRLEKLKEGIRDINKAKYLM
         BvGH123   1  NLDGYLRWALNSWVKNP...LQDSRF.....TAWAAGDTYMIYPGARSS........IRLERLTEGIQFFEKVRILK
         NgaP      1  QTDGYLRWAFDSFVKDP...YETTDF.....KTWESGDSAQVYPGAVSS........VRFERMKEGIRDAEKVRYLT
Group 1  NgaMg     1  GFSGILDWAADYVTKGQDP.WQDVSYL..ENKTVYQADGLLVYPGDKVGLPGTVVPSMRLKWLRDGVEDYEYVELLK
         NgaSc     1  GIKGVLYWRIDDWT..DEP.WEKVPVYV.HGEKNFPGEGMLVYRGTEVGLKT.IVPSMRLKWIREGVEDYEYIEILK
         NgaNp     1  GLTGILYWQVDLWT..KDP.WHDIQTYSKNNGDNFPGEGMLVYPGKQVGLQG.VIPSMRLKWLRDGVEDYEYMEIIK
         NgaCs     1  GLTGALYWRVDFWT..DDP.WNNVETLF.QDGNHYPGEGMLVYPGQQVGIEG.VVPSMRLKWLRDGVEDYEYIQILK
         NgaCa     1  GMTGILYWRVDFWT..EDP.WHDVLTLR.ADGMEFNGEGMLVYPGEQVGIDG.VVPSIRLKAIRKGIEDYEYIEILK
Group 2  NgaOs     1  GGTGFLYWGTNCYEKAM.....IPSAEICFRRGLPPGDGVLFYPGEVFSSSHEPVASTRLERILSGMQDIEYLKLYS
         NgaAt     1  GGTGFLYWGANCYEKAT.....VPSAEVKFRRGLPPGDGVLYYPGEVFSSSSEPVASLRLERLLSGLQDYEYLKLYE
         NgaGm     1  GGTGFLYWGANCYEKAT.....VASAEIKFRHGLPPGDGVLYYPGEVFSTSHQPVASLRLERILNGLQDTEYLRLYA
Group 4  NgaBl     1  DLAGFLHWGFNFYNAQYSLRPINPYTEAGTPEGFAAGDAFLVYPGP....GGAPEESIRIMVVEEALNDLRACRLLE
         NgaBf     1  DAPGFLHWGYNFWWAQFSLRPIDPFEETCAGGPFFGGDSFAVYPGP....EGTPWLSLRHRVFAQAMADHRALTWLA
         NgaLy     1  QIQGFLHWGFNFYNAQLSTRPIDPFAVTDAGGAFPSGDPFLVYPGA....DGQPLNSLRNEVQRLGFGDLAVLQQLE
         NgaP2     1  GVQGFLHWGFNFWYSQYSKKVIDPFKVTDADCAFPSGDPFVVYPGA....DG.PLDSIRWEVFREGLQDLRALKLLE
Group 3  NgaCp     1  DLTGFMHWGYNIWTGYP...FDFSTSN......SVGGDAWIVYPKD....GK.IISSVRLEAMRDGIVDYELLRLLE
         NgaDssm   1  GMTGYLHWGYNQWGKDS...PFTHTTKQHGQQYLPAGDPWIVYPGR....DG.PLDSIRHEAMCDGIADYELLSMLG
         NgaBa     1  GLVGYLHWGYNYWTEKP...FEDVENEPSHPFPLPPGDPFIVYPGK....SG.PLDSIRFEALRDGVEDYELFKLLA
```

c Sequence Identity (%) corresponding to DUF4091

GH123	CpNga123	100	43	46	29	26	23	29	27	30	30	27	25	22	25	28	31	25	34
	BvGH123	43	100	52	31	25	29	29	31	31	32	32	29	22	28	34	34	34	36
	NgaP	46	52	100	31	25	22	24	25	29	29	31	26	22	31	31	32	36	33
Group 1	NgaMg	29	31	31	100	51	59	59	55	30	34	31	21	21	24	26	30	28	40
	NgaSc	26	25	25	51	100	60	61	58	29	31	29	21	21	24	24	28	24	36
	NgaNp	23	29	22	59	60	100	69	67	29	33	30	22	23	25	25	33	30	40
	NgaCs	29	29	24	59	61	69	100	72	31	34	31	26	25	28	28	33	32	42
	NgaCa	27	31	25	55	58	67	72	100	32	32	32	25	28	31	34	33	38	38
Group 2	NgaOs	30	31	29	30	29	29	31	32	100	83	79	31	29	34	36	31	34	37
	NgaAt	30	32	29	34	31	33	34	32	83	100	85	34	28	37	39	34	36	40
	NgaGm	27	32	31	31	29	30	31	32	79	85	100	32	28	35	34	33	34	37
Group 4	NgaBl	25	29	26	21	21	22	26	25	31	34	32	100	51	49	51	35	32	35
	NgaBf	22	22	22	21	21	23	25	28	29	28	28	51	100	52	50	32	35	36
	NgaLy	25	28	31	24	24	25	28	31	34	37	35	49	52	100	67	32	38	38
	NgaP2	28	34	31	26	24	25	28	34	36	39	34	51	50	67	100	35	42	46
Group 3	NgaCp	31	34	32	30	28	33	33	33	31	34	33	35	32	32	35	100	48	48
	NgaDssm	25	34	36	28	24	30	32	38	34	36	34	32	35	38	42	48	100	52
	NgaBa	34	36	33	40	36	40	42	38	37	40	37	35	36	38	46	48	52	100

Supplementary Figure 3. Candidate β-NGA sequences containing DUF4091.
a, Sequence identities within β-NGA genes. Names in red represent the enzymes whose activities were experimentally confirmed in this study. **b**, Alignment of the gene sequences corresponding to DUF4091. **c**, Sequence identities corresponding to DUF4091 with known GH123 and β-NGA candidates.

a

Sequence Identity (%)

	NgaAt	HEXO2	HEXO1	HEXO3
NgaAt	100.0	17.2	19.3	18.7
HEXO2	17.2	100.0	35.0	33.1
HEXO1	19.3	35.0	100.0	51.8
HEXO3	18.7	33.1	51.8	100.0

b

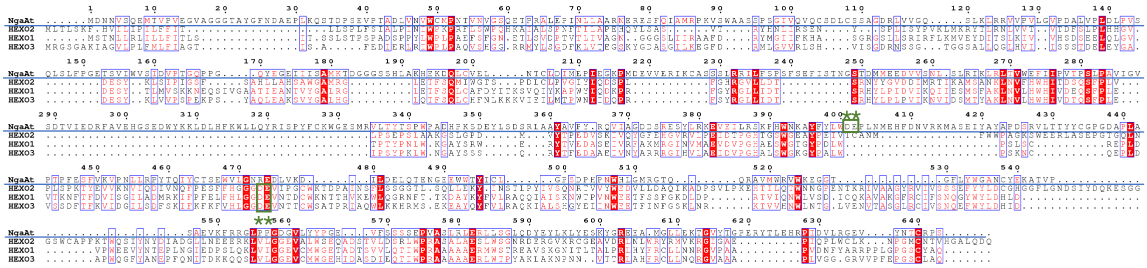

c

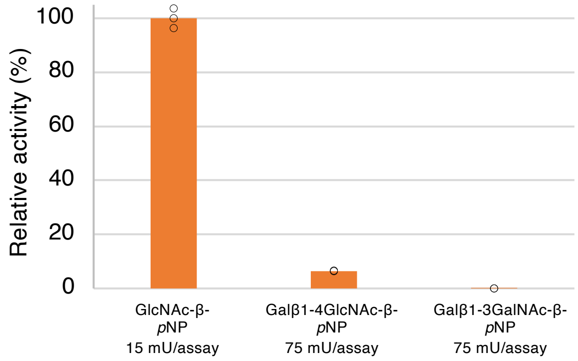

Supplementary Figure 4. Comparison between NgaAt and HEXO1–3.
a, Sequence identities within the *NgaAt* and *HEXO1–3* genes. **b**, Alignment of gene sequences. Residues conserved in all the proteins are displayed in a red background. The DE motifs (green asterisk, *) of NgaAt and HEXO1–3 are indicated by green boxes. **c**, Specificity of NgaAt against different *p*NP substrates. Values represent the mean of technical triplicate measurements.

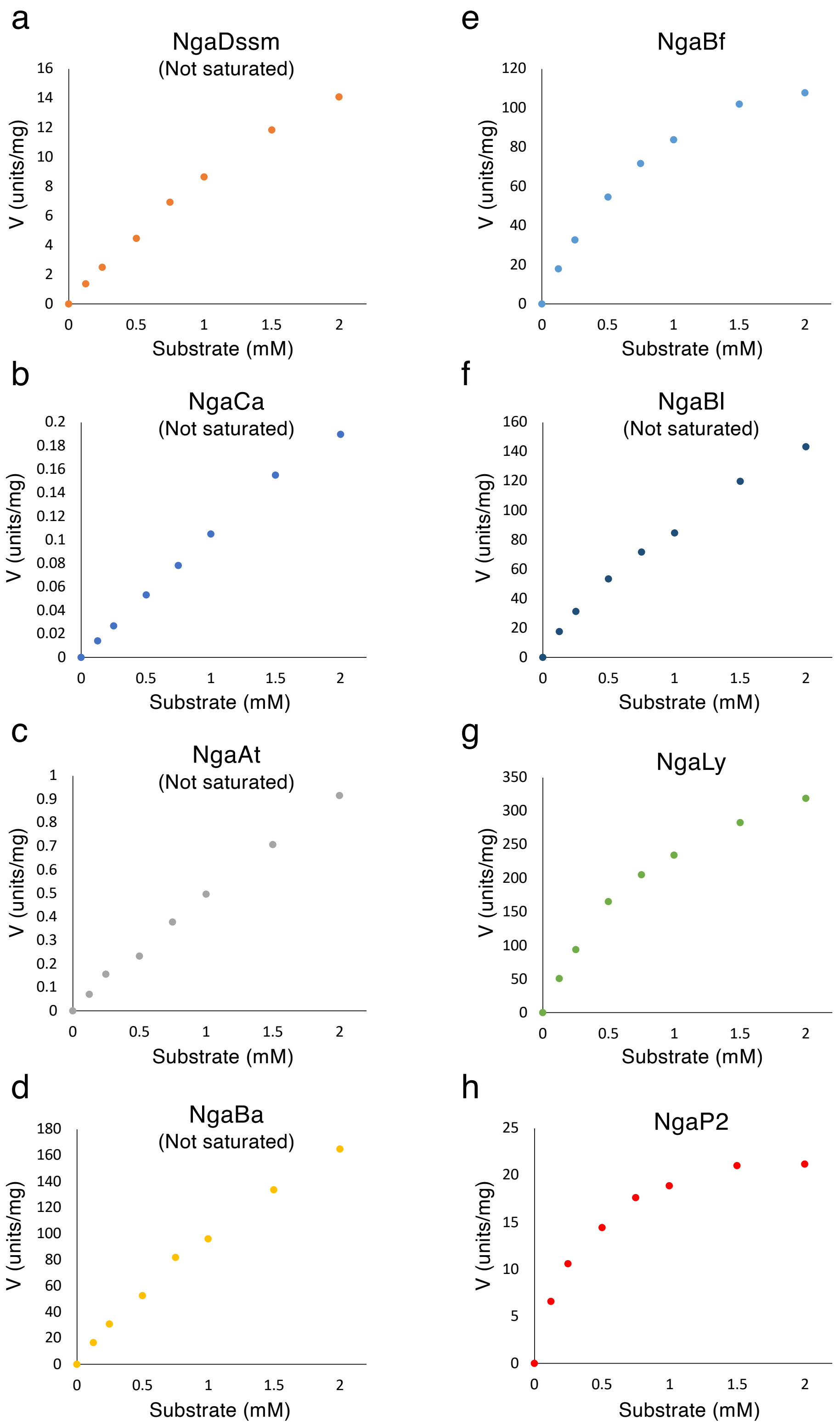

Supplementary Figure 5. s-v plots of recombinant β-NGAs. All values represent the mean of triplicate measurements.

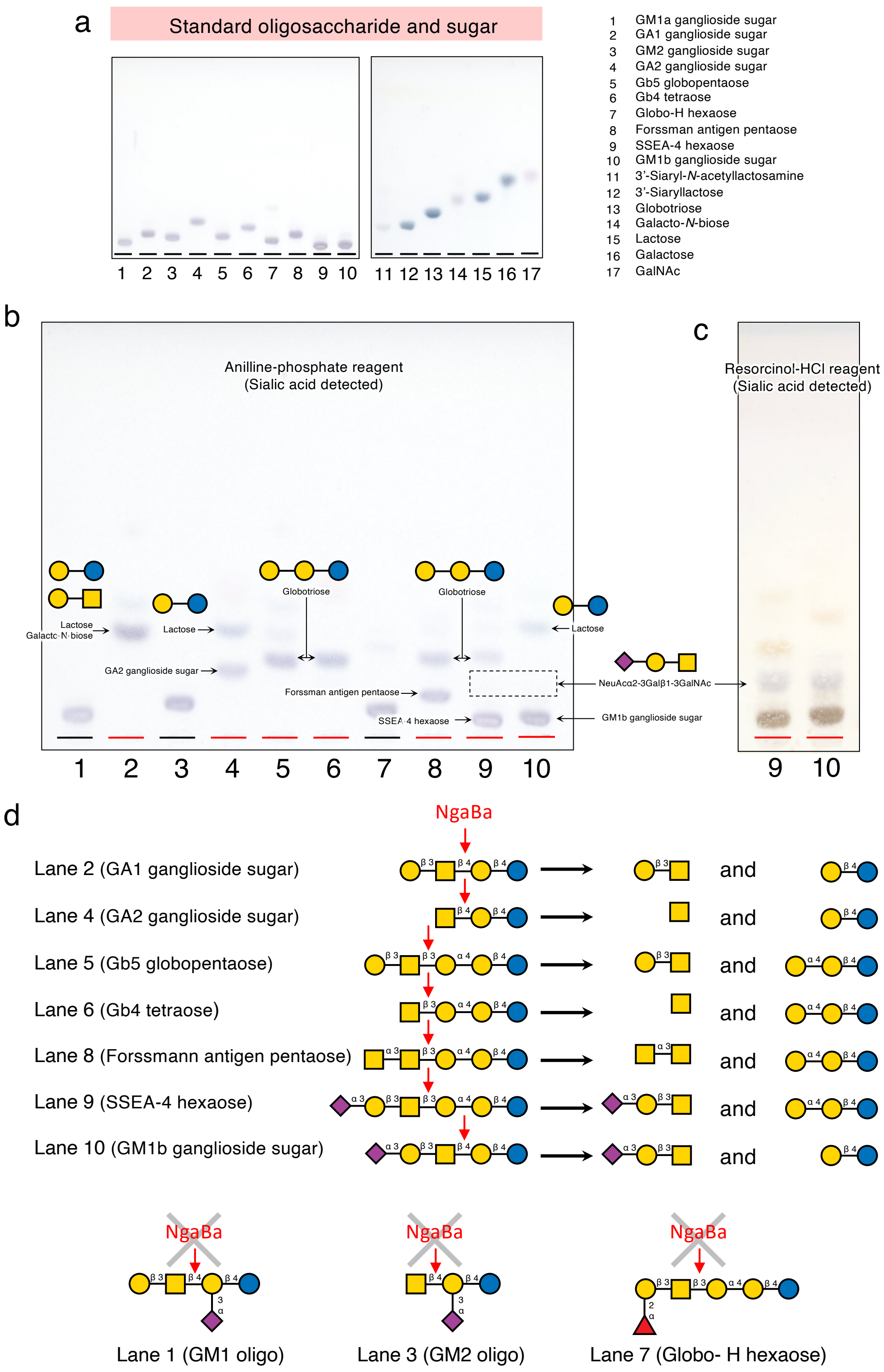

Supplementary Figure 6. Hydrolysis of oligosaccharides by recombinant β-NGAs.
a, Thin layer chromatography analysis of the standard oligosaccharides and sugars. The Rf values of the standard oligosaccharides and sugar are listed in the rightmost column. **b**, **c,** Hydrolysis of oligosaccharides by recombinant NgaBa. Oligosaccharides in each lane are arranged in the same order as in the standard TLC. Oligosaccharides and sugar are detected with diphenylamine-aniline-phosphate reagent (**c**) and resorcinol-HCl reagent (**d**), respectively. **e**, Symbol nomenclature for the GalNAc-containing glycans used in this experiment and cleavage site of each oligosaccharide by NgaBa.

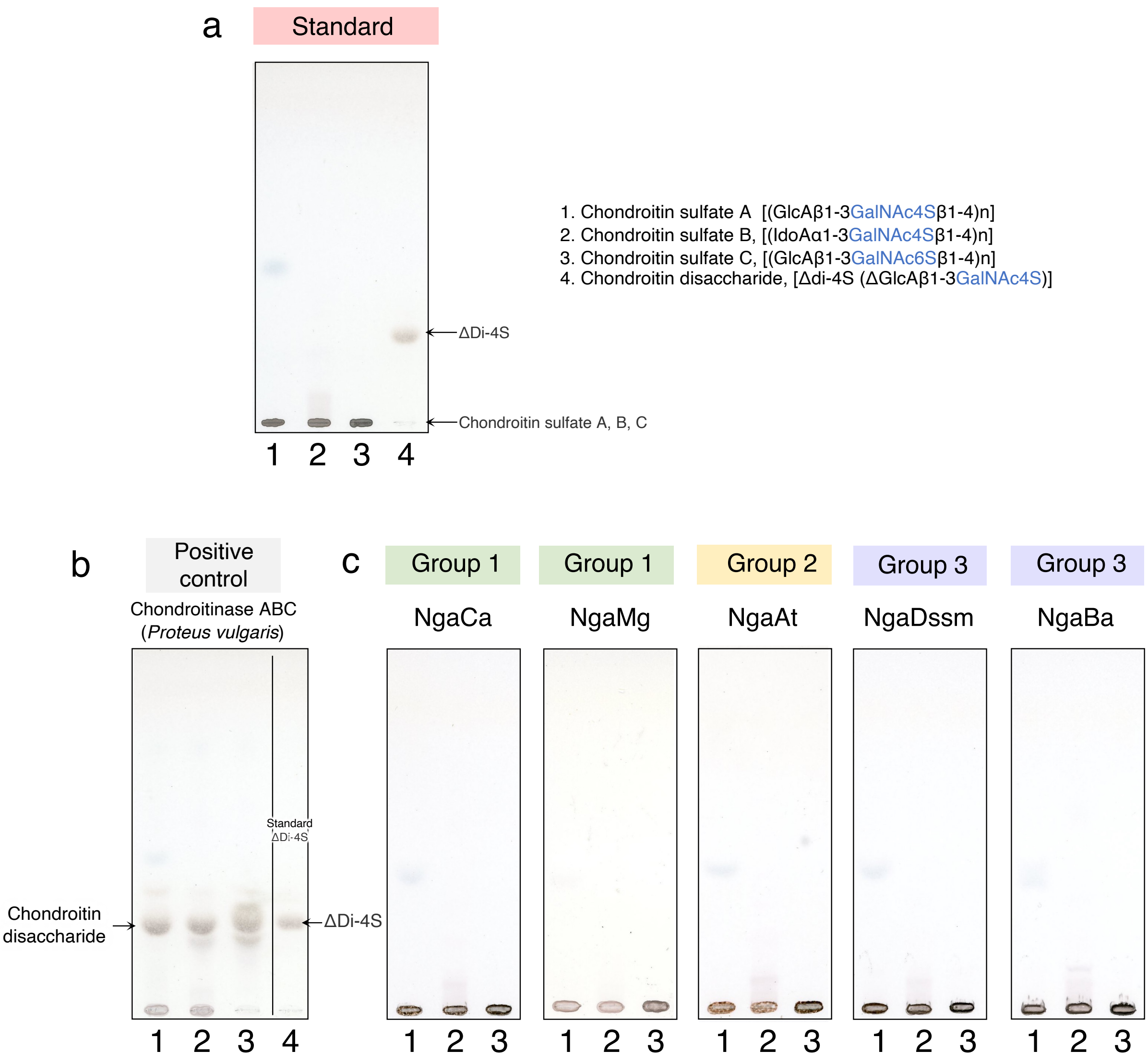

Supplementary Figure 7. Hydrolysis of chondroitin sulfate A, B, C by recombinant β-NGAs.
a, Thin layer chromatography analysis of the standard chondroitin sulfate and disaccharide (ΔDi-4S). **b**, Hydrolysis of chondroitin sulfates by recombinant chondroitinase ABC (EC 4.2.2.4) from *Proteus vulgaris* as a control assay. **c,** Hydrolysis of chondroitin sulfates by recombinant β-NGAs. chondroitin sulfates in each lane are arranged in the same order as in the standard TLC. Oligosaccharides and sugar are detected with diphenylamine-aniline-phosphate reagent.

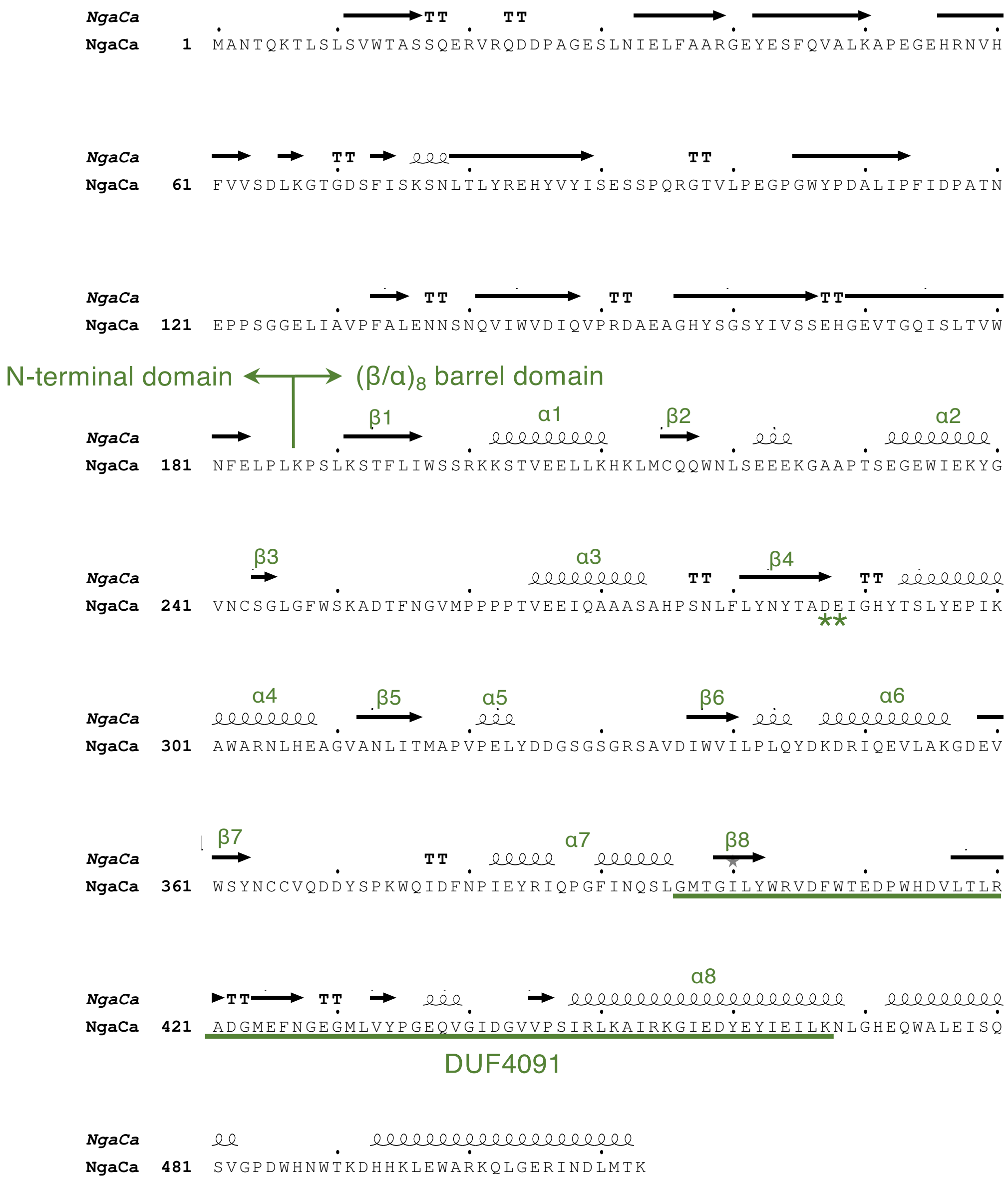

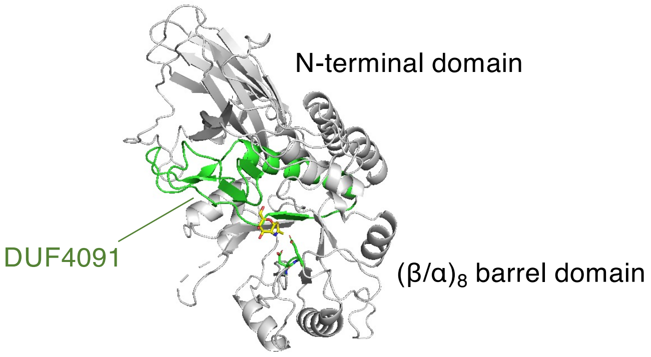

Supplementary Figure 8. Secondary structure and characteristic domains of NgaCa (Group 1). The secondary structural elements are indicated above the sequence. The $(\beta/\alpha)_8$-barrel region is indicated above the secondary structural elements. The conserved DE residues (*) are indicated below the sequence. The DUF4091 region is underlined.

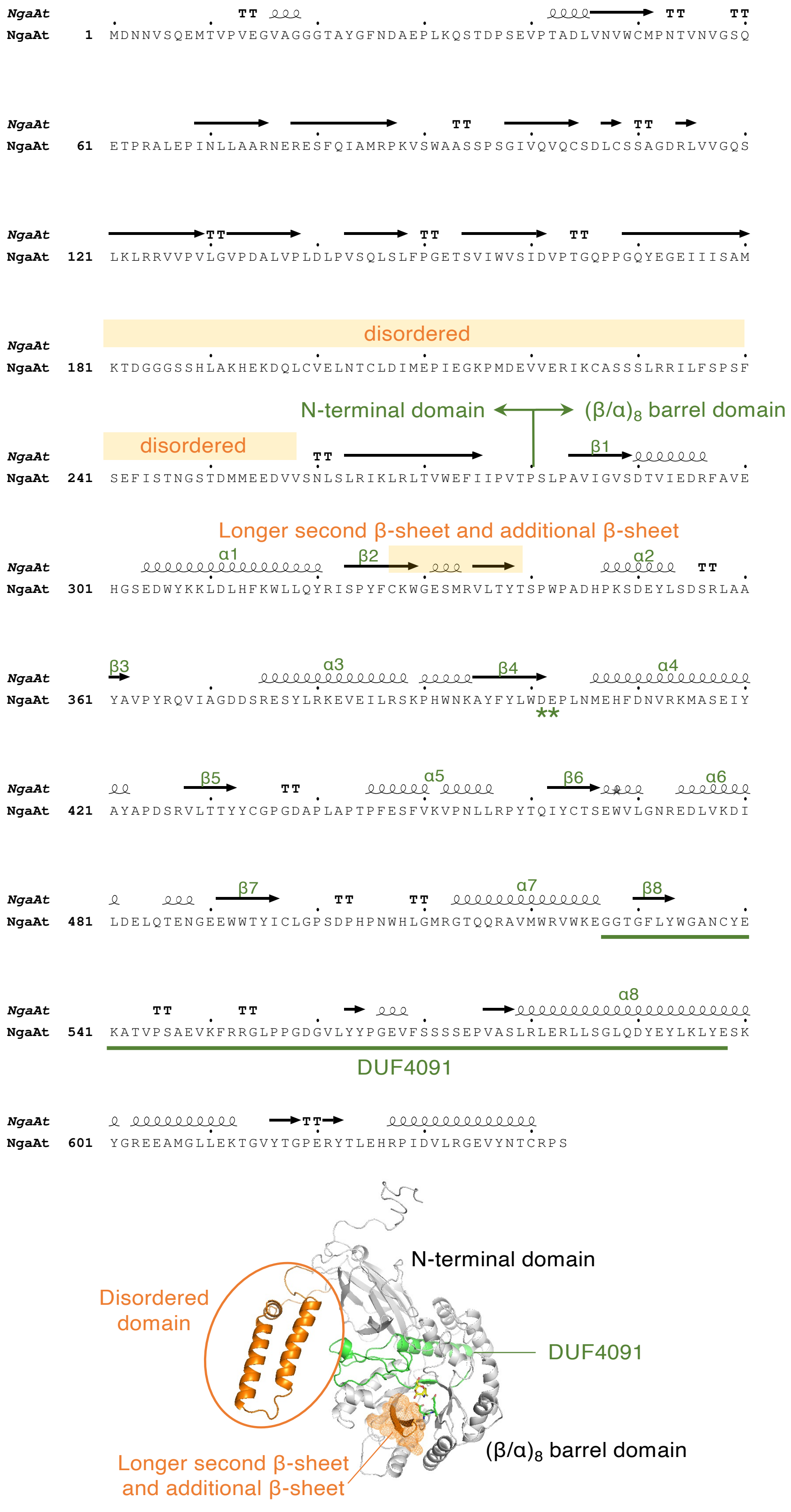

Supplementary Figure 9. Secondary structure and characteristic domains of NgaAt (Group 2). The secondary structural elements are indicated above the sequence. The additional domain characteristic of Group 2 is shown in orange. The $(\beta/\alpha)_8$-barrel region is indicated above the secondary structural elements. The conserved DE residues (*) are indicated below the sequence. The DUF4091 region is underlined.

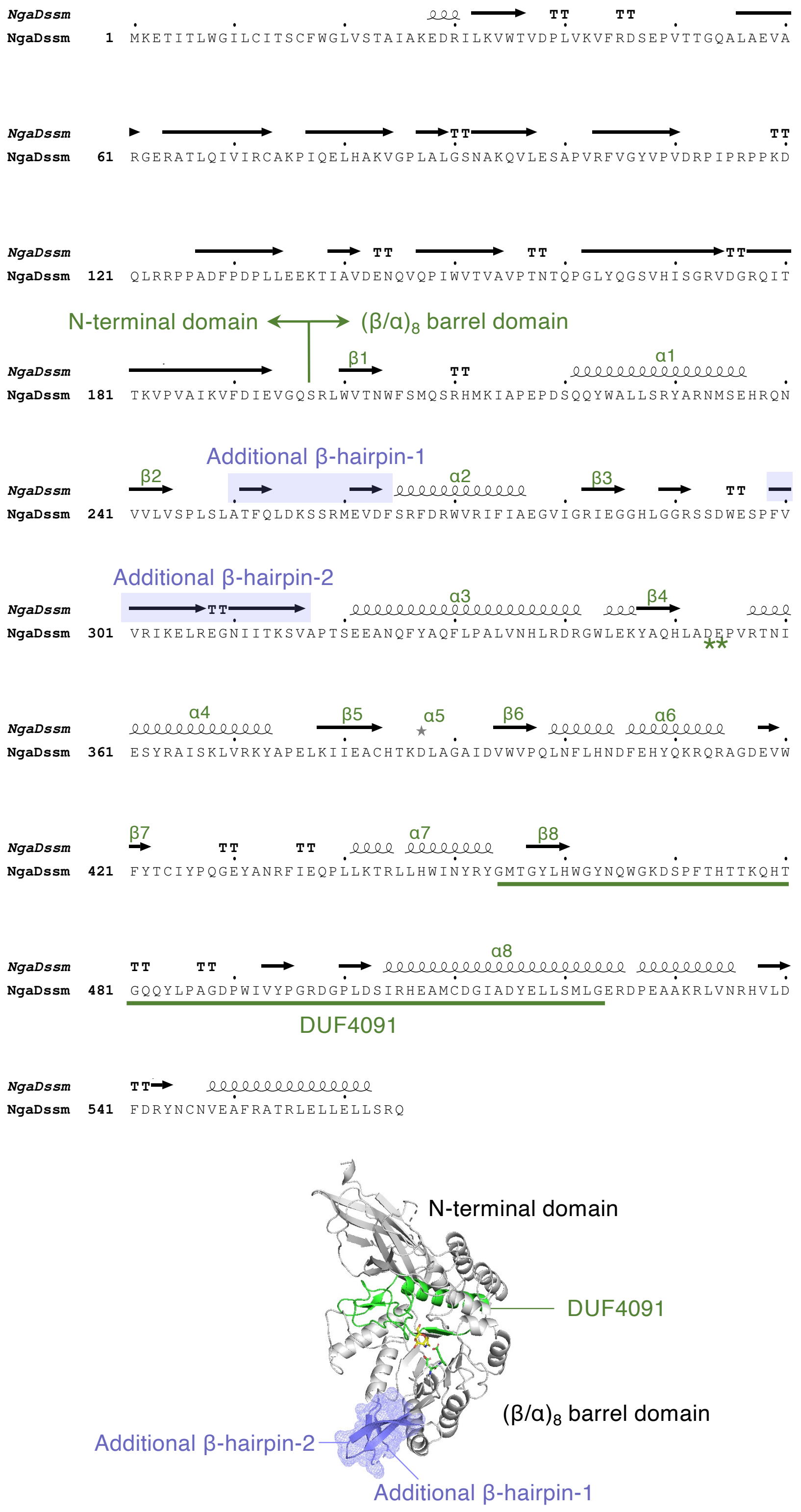

Supplementary Figure 10. Secondary structure and characteristic domains of NgaDssm (Group 3). The secondary structural elements are indicated above the sequence. The additional domain characteristic of Group 3 is shown in purple. The $(\beta/\alpha)_8$-barrel region is indicated above the secondary structural elements. The conserved DE residues (*) are indicated below the sequence. The DUF4091 region is underlined.

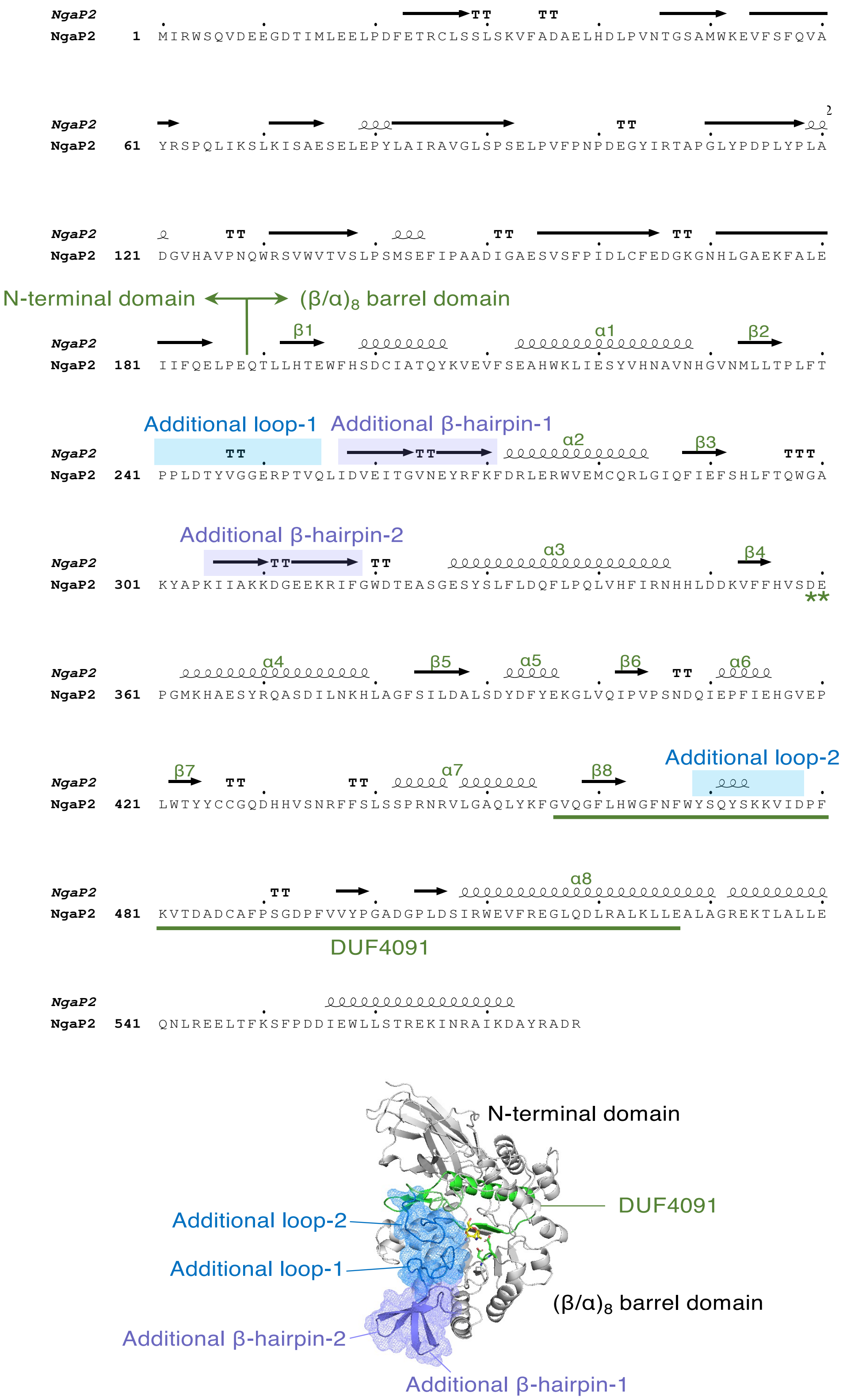

Supplementary Figure 11. Secondary structure and characteristic domains of NgaP2 (Group 4). The secondary structural elements are indicated above the sequence. The additional β-hairpins and loops characteristic of Group 4 are shown in purple and blue, respectively. The (β/α)$_8$-barrel region is indicated above the secondary structural elements. The conserved DE residues (*) are indicated below the sequence. The DUF4091 region is underlined.

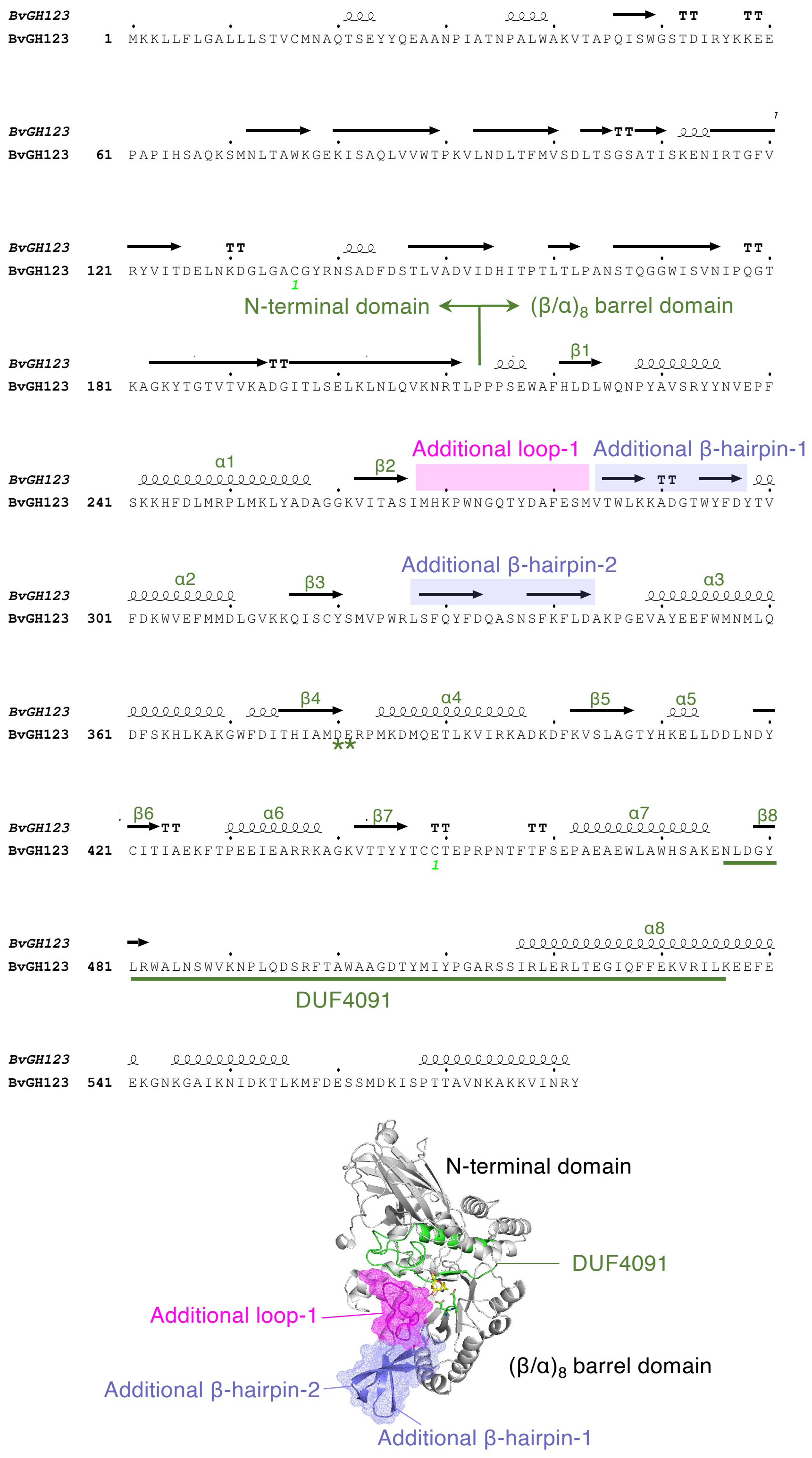

Supplementary Figure 12. Secondary structure and characteristic domains of BvGH123 (GH123). The secondary structural elements are indicated above the sequence. The additional β-hairpins and loops characteristic of GH123 are shown in purple and magenta, respectively. The $(\beta/\alpha)_8$-barrel region is indicated above the secondary structural elements. The conserved DE residues (*) are indicated below the sequence. The DUF4091 region is underlined.

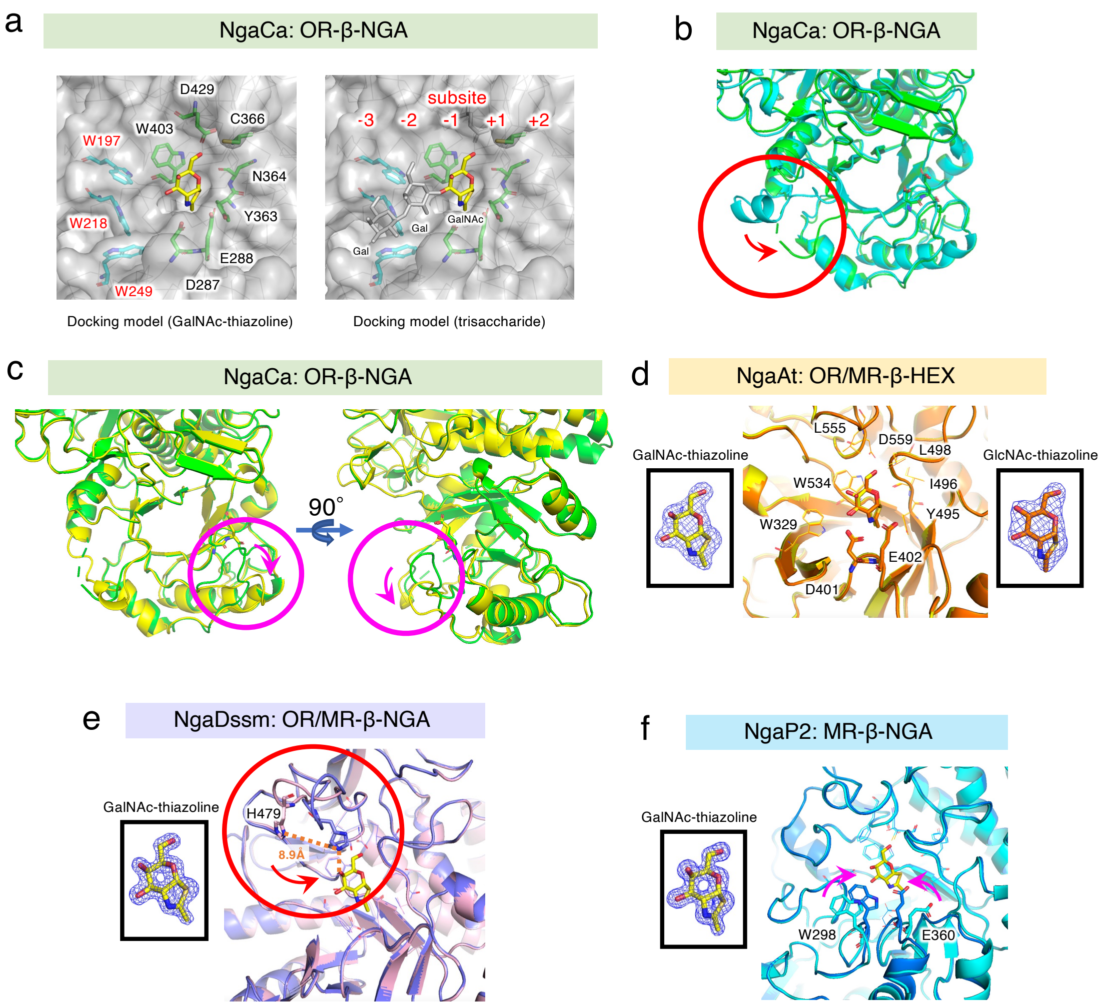

Supplementary Figure 13. The structures of NgaCa, NgaAt, NgaDssm, and NgaP2.
a, Characteristic tryptophan on the subsite minus area of NgaCa. Docking models of NgaCa are constructed with GalNAc-thiazoline and trisaccharide. GalNAc-thiazoline is manually docked, referring to the complex structure of the NgaDssm and GalNAc-thiazoline, and trisaccharide is manually docked referring to the shape of the cleft form. GalNAc-thiazoline recognition residues and characteristic tryptophan are shown as green and cyan sticks, respectively. **b,** Comparison between the apo1 crystal structure (green) and AlphaFold2-predicted structure (cyan) of NgaCa. The position of the loop behind the second β-sheet in the $(\beta/\alpha)_8$-barrel domain is demarcated with a red circle. The DE motif is indicated by a stick. **c**, Distinction between apo 1 (green) and apo 2 (yellow) in NgaCa. The apo 2 structure is derived from crystals to which 5 mM Galβ1-3GalNAc is incorporated during crystallization, but Galβ1-3GalNAc is not visible. However, the loops surrounding the active site are different from those surrounding apo 1, and the loops move as the active site cleft expands (magenta circle). These two distinct states are designated as closed (apo 1) and open (apo 2), respectively. The DE motif is indicated by a stick. **d**, Superimposition of the GalNAc- and GlcNAc-thiazoline bond forms of NgaAt (yellow and orange, respectively). The positions of the amino acids involved in substrate recognition are identical. GalNAc- and GlcNAc-thiazoline are represented by yellow and orange sticks, respectively. Polder maps of GalNAc- and GlcNAc-thiazoline (4σ) are illustrated as blue meshes. **e**, Superimposition of the apo (pink) and the GalNAc-thiazoline-bound forms (purple) of NgaDssm. In the apo form, His479 is distant from the active site. In the GalNAc-thiazoline bond, His 479 is located within hydrogen-bonding distance from the 4-OH of GalNAc-thiazoline. These two distinct forms are designated as closed and open states, respectively. GalNAc-thiazoline is depicted by a yellow stick. A polder map of GalNAc-thiazoline (4σ) is displayed as a blue mesh. **f**, Superimposition of the apo form (cyan) of NgaP2 onto its GalNAc-thiazoline-bound form (blue). The residues that shifted upon GalNAc-thiazoline binding, Trp298 and Glu360, are depicted as sticks. The displacements of these residues are indicated by arrows. GalNAc-thiazoline is represented by a yellow stick. A polder map of GalNAc-thiazoline (4σ) is illustrated as a blue mesh.

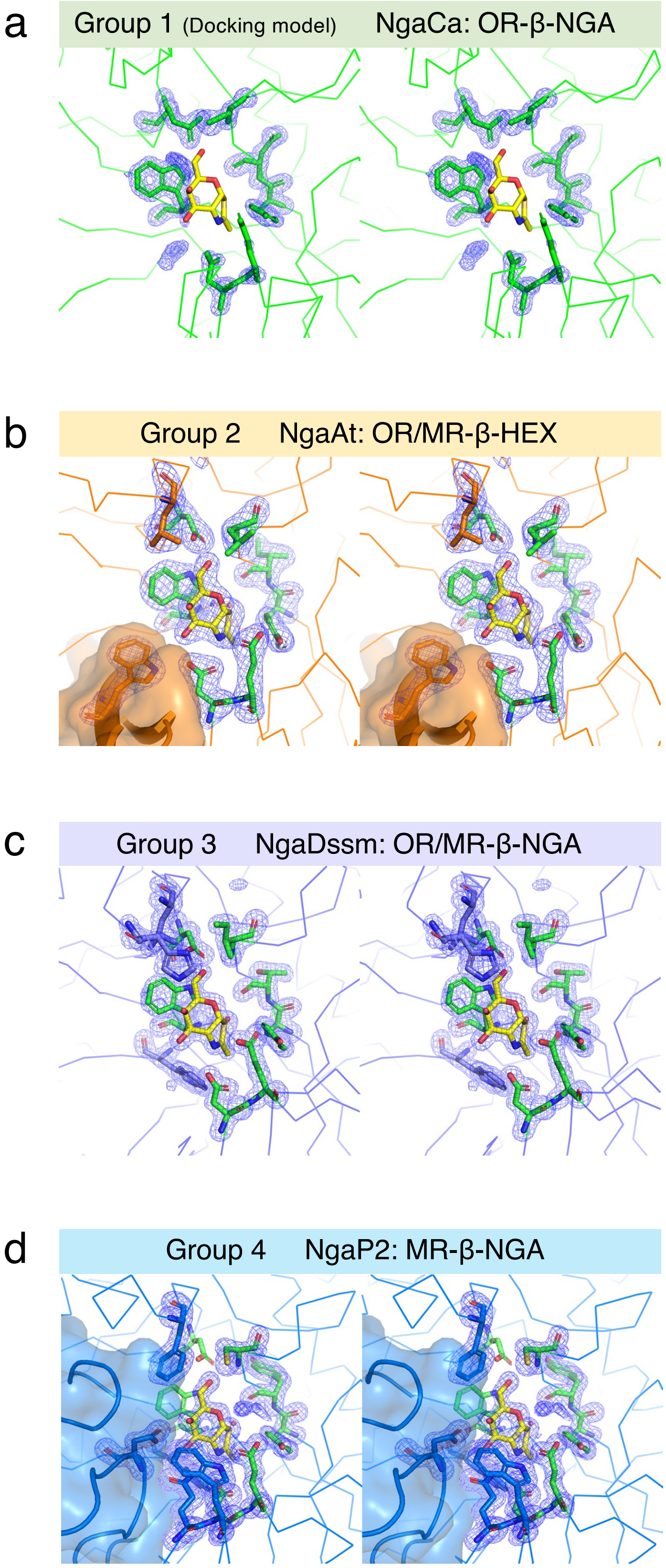

Supplementary Figure 14. Stereo view of the active site of β-NGAs.
a, A docking model of NgaCa with GalNAc-thiazoline. **b–d**, Crystal structures of each enzyme complexed with GalNAc-thiazoline. Amino acid residues and GalNAc-thiazoline are shown in stick. Polder maps (4σ) are shown as blue mesh.

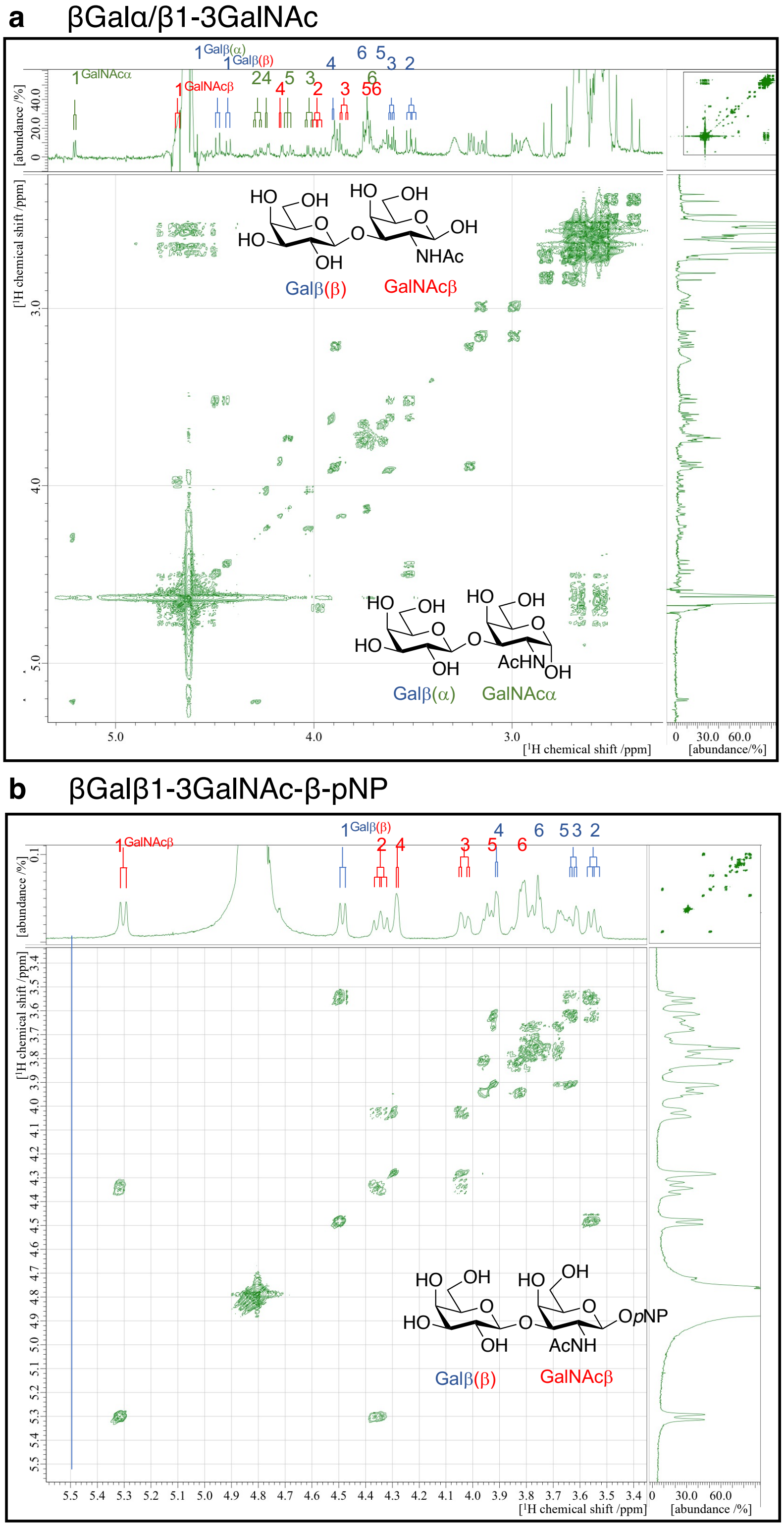

Supplementary Figure 15. 2D ^{1}H-^{1}H COSY spectra of βGalα/β1-3GalNAc and βGalβ1-3GalNAc-β-*p*NP.
a, 2D ^{1}H-^{1}H COSY spectrum of reaction mixture under equilibrium after hydrolysis followed by mutarotation. The mixture contains approximately 1.5:1 mixture βGalα/β1-3GalNAc. Assignments are conducted by 1D ^{1}H NMR and 2D ^{1}H-^{1}H COSY. Since the chemical shift of ^{1}H at 1 of β-GalNAc (4.58 ppm) is overlapped under large HOD peak (4.4–4.8). **b**, 2D ^{1}H-^{1}H COSY spectrum of βGalβ1-3GalNAc-*p*NP in D_2O. Assignments are conducted by 1D ^{1}H and ^{13}C, and 2D ^{1}H-^{1}H COSY, TOCSY HMQC, and HMBC.

a

b

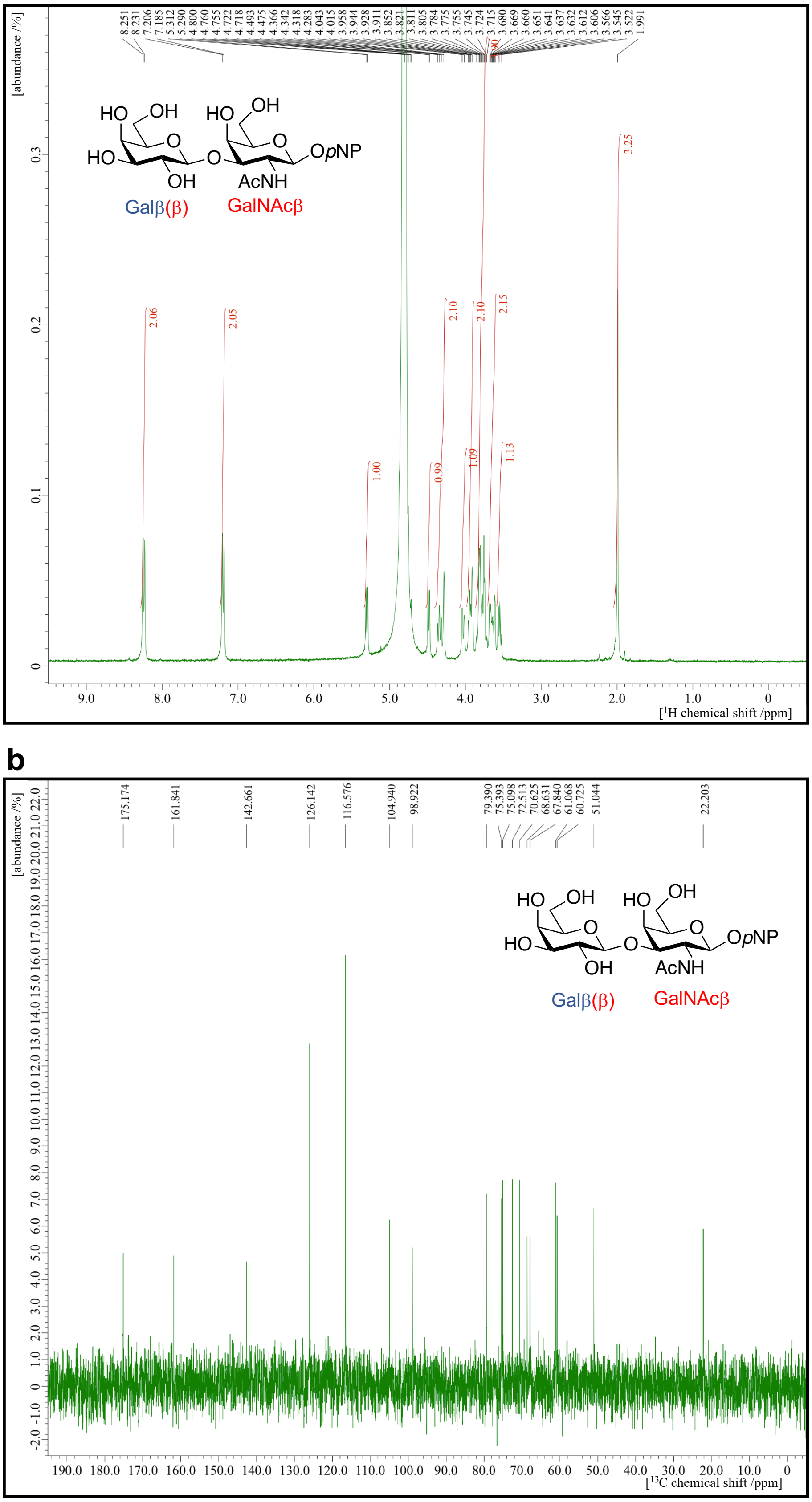

Supplementary Figure 16. NMR spectra of Galβ1-3GalNAc-β-*p*NP in D_2O.
a, ^{1}H NMR spectrum, **b**, ^{13}C NMR spectrum.

c

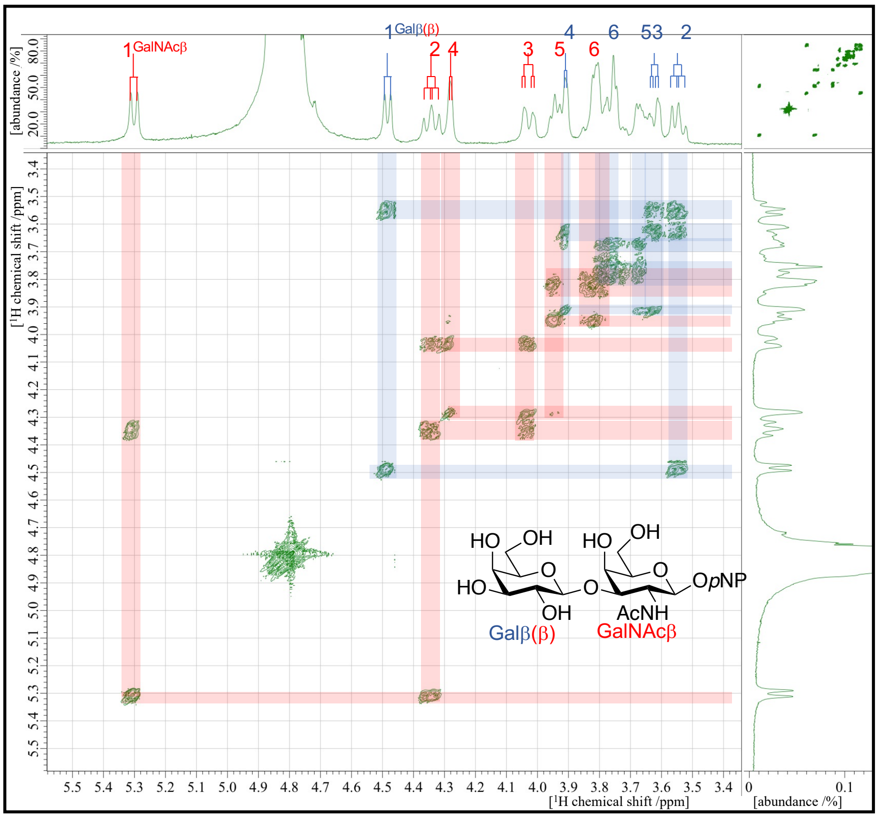

d

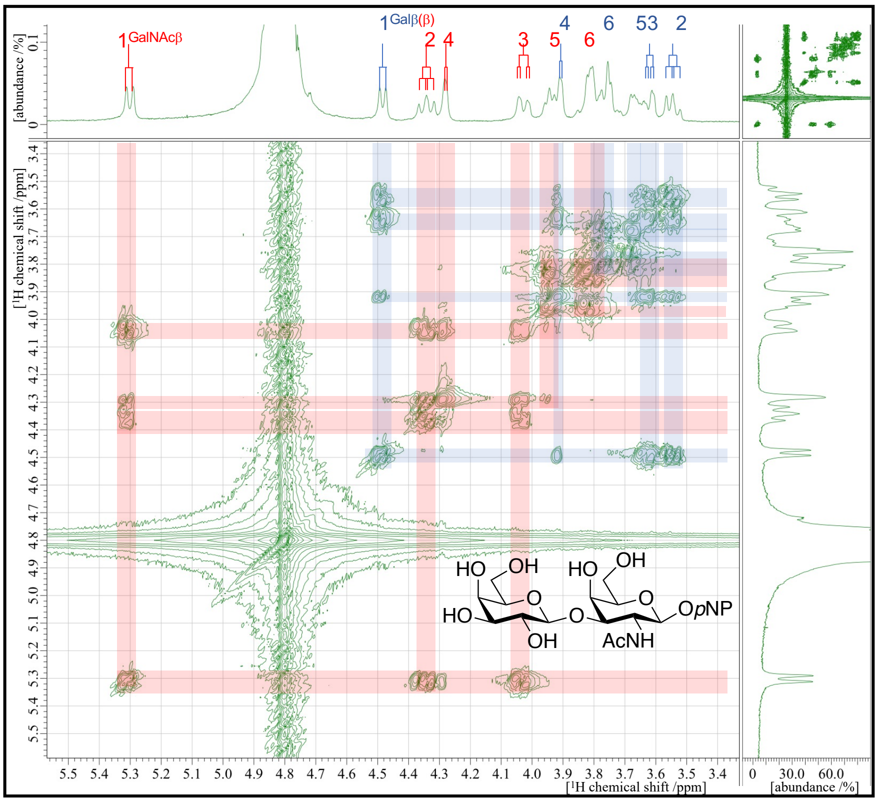

Supplementary Figure 16. NMR spectra of Galβ1-3GalNAc-β-*p*NP in D_2O.
c, 2D ^{1}H–^{1}H COSY spectrum, **d**, TOCSY spectrum. Assignments are shown with the multiplications and the position numbers in each residue on the top.

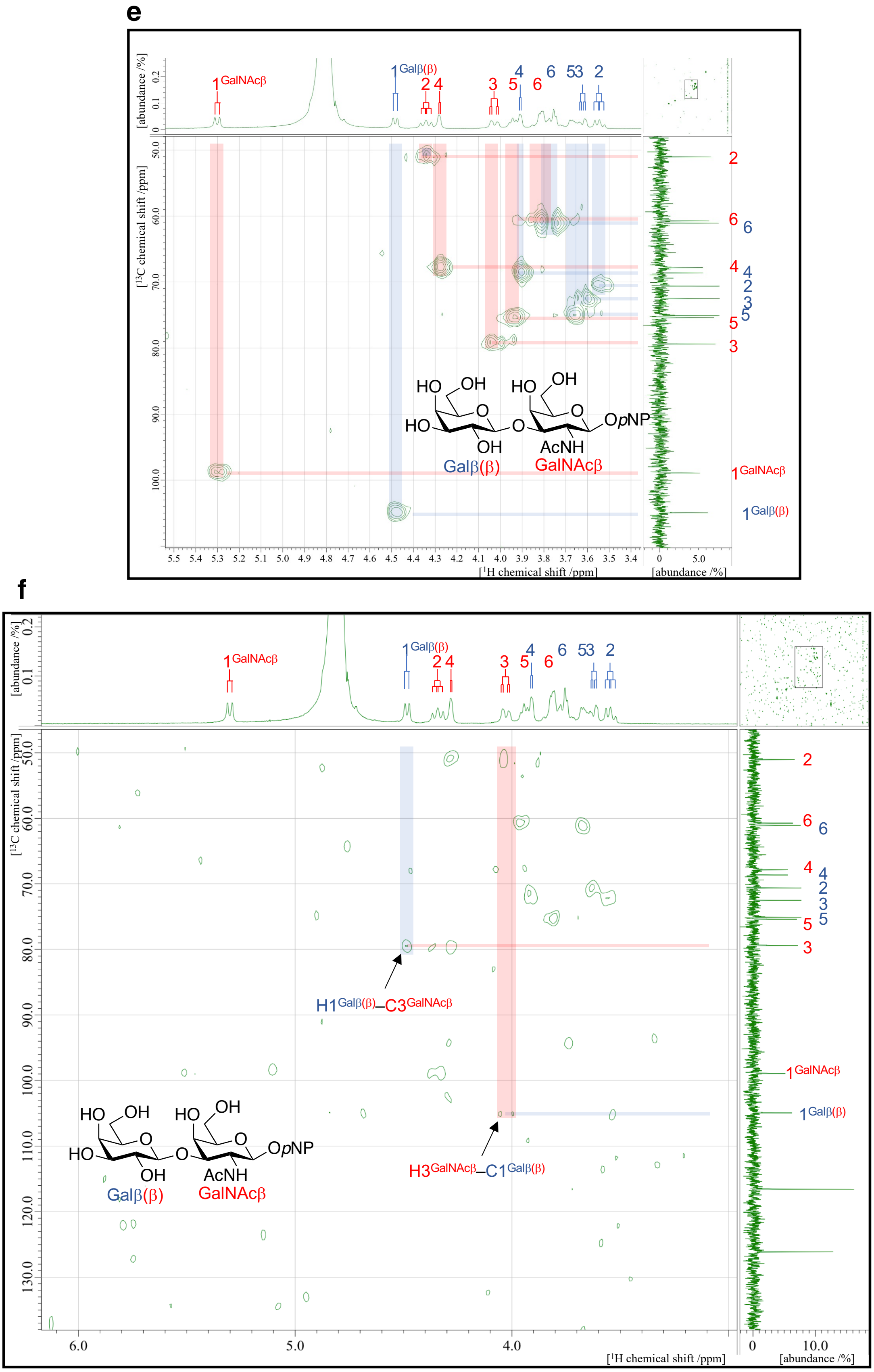

Supplementary Figure 16. NMR spectra of Galβ1-3GalNAc-β-*p*NP in D_2O.
e, HMQC spectrum, **f**, HMBC spectrum. Assignments are shown with the multiplications for ^{1}H NMR and the position numbers for both ^{1}H and ^{13}C NMRs in each residue.

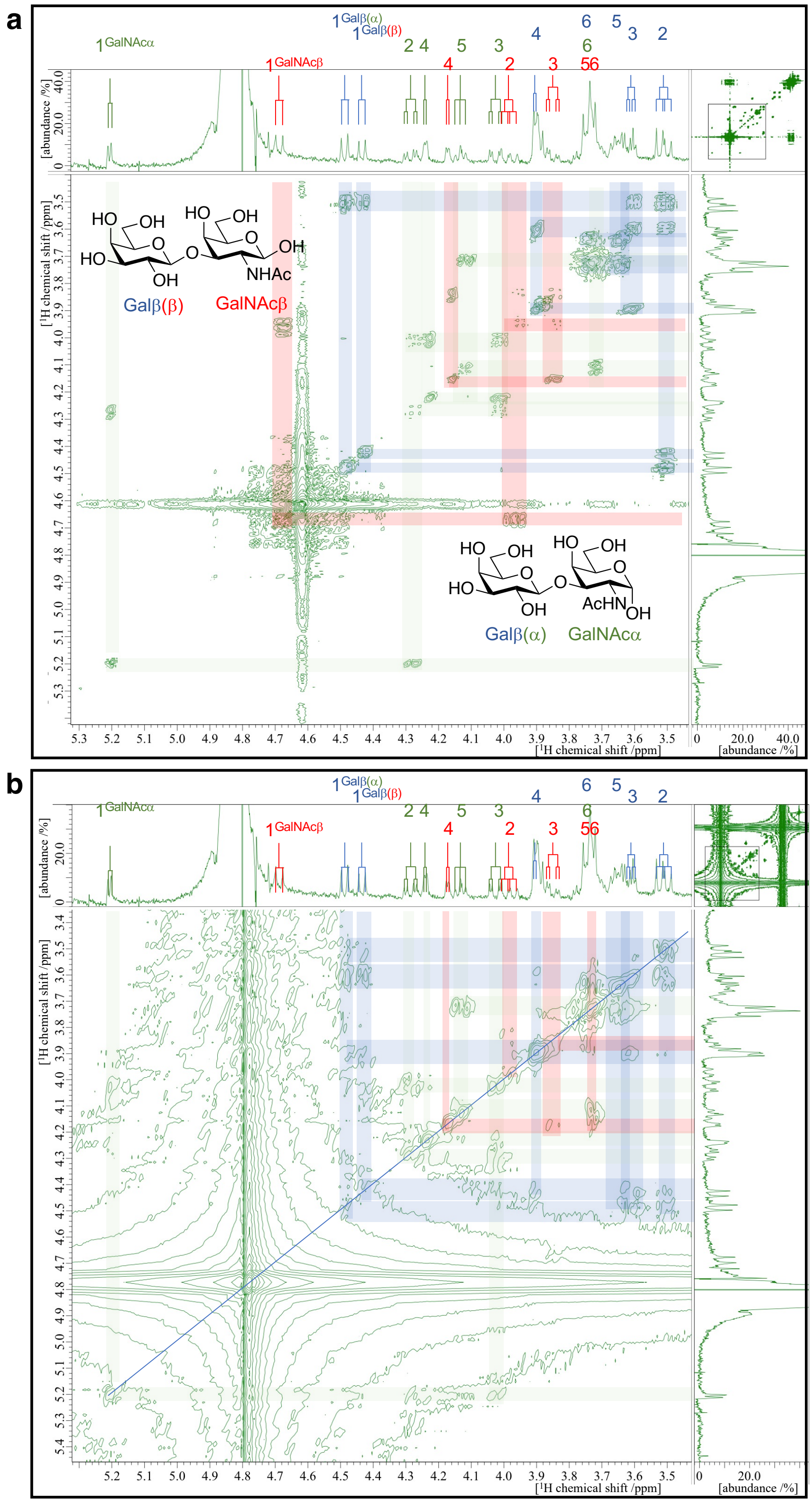

Supplementary Figure 17. NMR spectra of Galβ1-3GalNAc-β-*p*NP treated with the enzyme at pH in D_2O.

a, 2D ^{1}H–^{1}H COSY spectrum, **b**, TOCSY spectrum. The reaction mixtures after 30 min and 24 h are used for the experiments. The same ^{1}H NMR spectra of the reaction mixture after 24 h is used for 2D ^{1}H–^{1}H COSY spectrum. The measurement of the sample after 24 h is conducted at room temperature. Assignments are shown with the multiplications and the position numbers of each residue on the top.

c

d

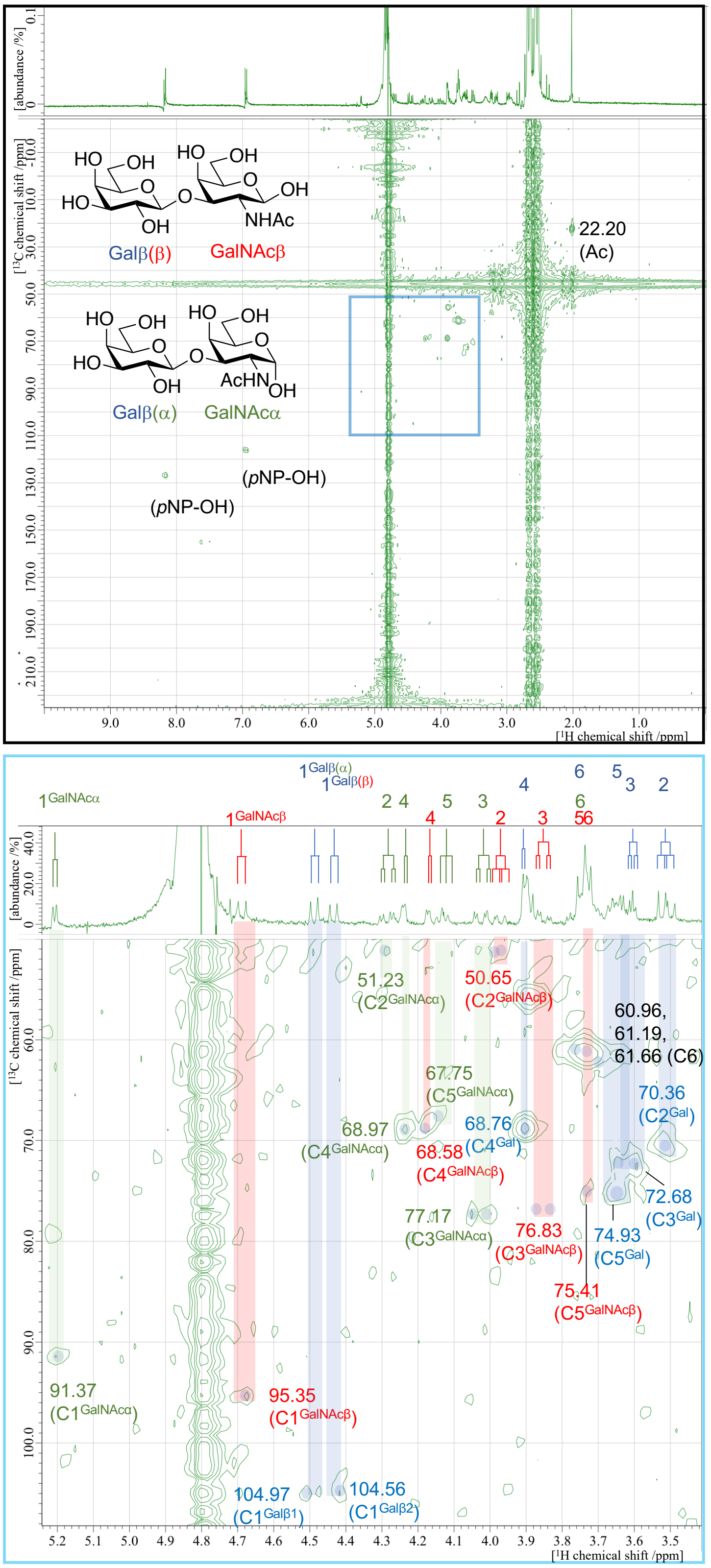

Supplementary Figure 17. NMR spectra of Galβ1-3GalNAc-β-*p*NP treated with the enzyme at pH in D_2O.

c, HMQC spectrum, **d**, ^{13}C assignment using HMQC spectrum. The reaction mixture after 24 h is used for the experiments. Assignments are shown with the multiplications for ^{1}H NMR and the position numbers for both ^{1}H and ^{13}C NMRs of each residue.

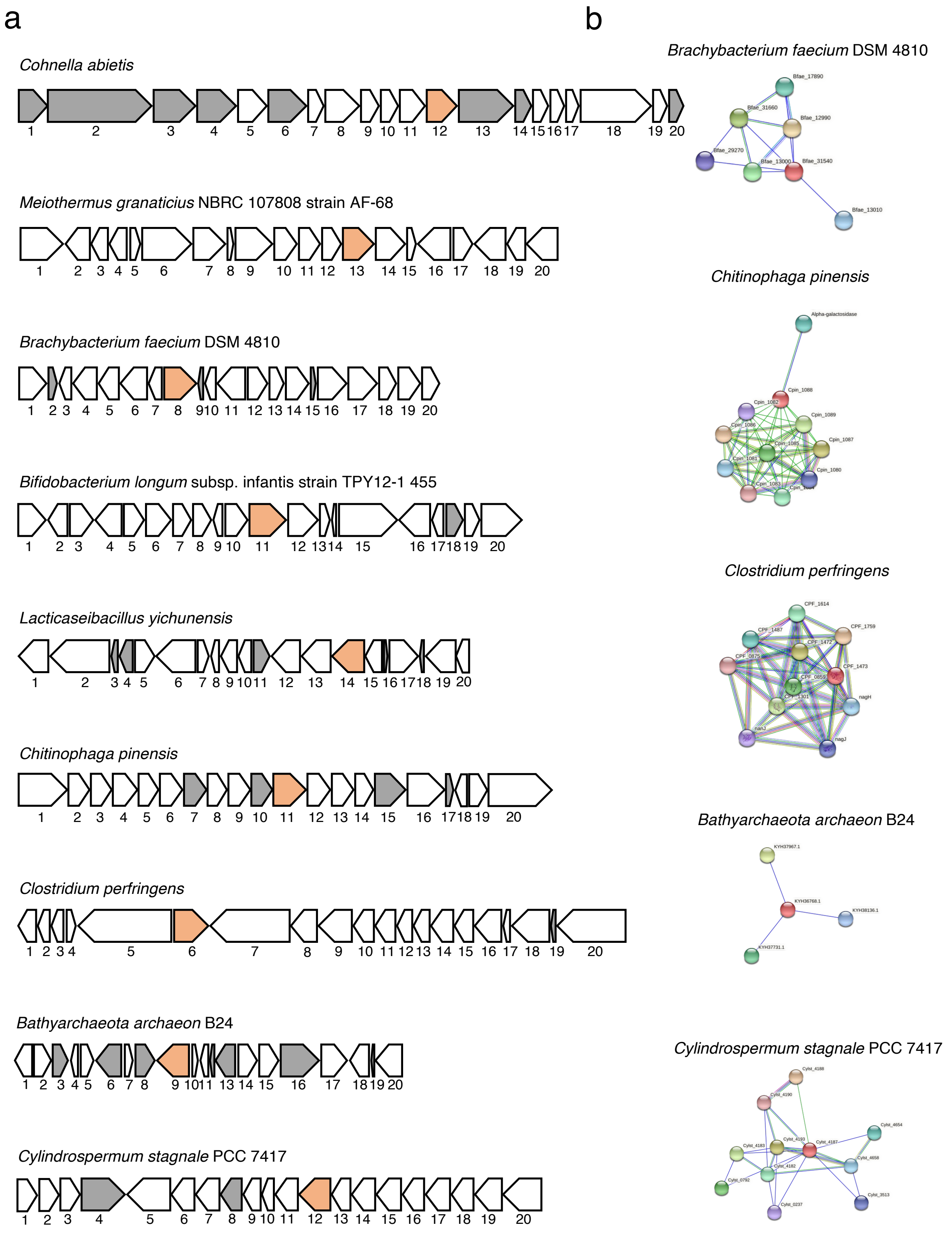

Supplementary Figure 18. Neighborhood genes and potential protein interaction networks of β-NGAs.

a, Neighborhood genes around the β-NGA. Functional annotations are obtained from the NCBI GenBank database, as listed in Supplementary Data S4. The β-NGAs and hypothetical proteins are depicted in orange and gray, respectively. **b**, Interaction networks of β-NGA retrieved from the STRING database (v11.5). Predicted functional partner proteins are listed in Supplementary Data S5.

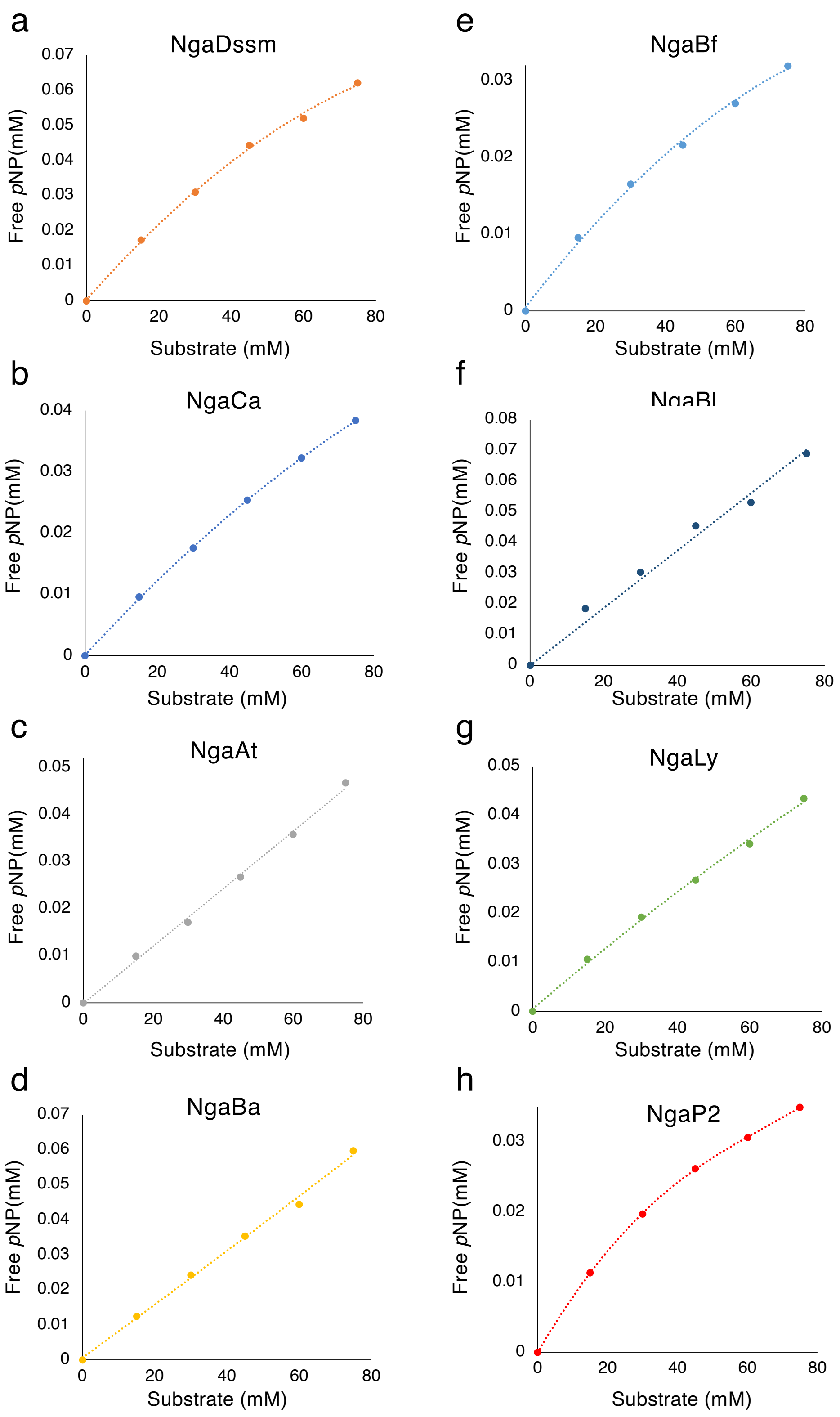

Supplementary Figure 19. Time course for the hydrolysis of GalNAc-β-*p*NP (or Galβ1-3GalNAc-β-*p*NP for NgaCa) by recombinant β-NGAs.

Supplementary Table 1. The structures and the Rf values of the standard oligosaccharides and sugars

	Substrate	Structure	Rf
1	GM1a ganglioside sugar	Galβ1-3GalNAcβ1-4(NeuAcα2-3)Galβ1-4Glc	0.06
2	GA1 ganglioside sugar	Galβ1-3GalNAcβ1-4Galβ1-4Glc	0.10
3	GM2 ganglioside sugar	GalNAcβ1-4(NeuAcα2-3)Galβ1-4Glc	0.08
4	GA2 ganglioside sugar	GalNAcβ1-4Galβ1-4Glc	0.14
5	Gb5 globopentaose	Galβ1-3GalNAcβ1-3Galα1-4Galβ1-4Glc	0.08
6	Gb4 tetraose	GalNAcβ1-3Galα1-4Galβ1-4Glc	0.13
7	Globo-H hexaose	Fucα1-2Galβ1-3GalNAcβ1-3Galα1-4Galβ1-4Glc	0.06
8	Forssman antigen pentaose	GalNAcα1-3GalNAcβ1-3Galα1-4Galβ1-4Glc	0.09
9	SSEA-4 hexaose	NeuAcα2-3Galβ1-3GalNAcβ1-3Galα1-4Galβ1-4Glc	0.04
10	GM1b ganglioside sugar	NeuAcα2-3Galβ1-3GalNAcβ1-4Galβ1-4Glc	0.04
11	3'-Siaryl-*N*-acetyllactosamine	NeuAcα2-3Galβ1-3GlcNAc	0.16
12	3'-Sialyllactose	NeuAcα2-3Galβ1-3Glc	0.18
13	Globotriose	Galα1-4Galβ1-4Glc	0.19
14	Galacto-*N*-biose	Galβ1-3GalNAc	0.24
15	Lactose	Galβ1-3Glc	0.30
16	Galactose	Gal	0.41
17	GalNAc	GalNAc	0.43

Supplementary Table 2. Data collection and refinement statistics of the crystallography of NgaCa (Group 1)

Data set	NgaCa apo 1	NgaCa apo 2 (GNB soaked)
**Data collection**		
Beamline	BL32XU	BL32XU
Wavelength (Å)	1.0000	1.0000
Space group	$P2_1$	$P1$
Unit cell (Å/°)	a = 48.7, b = 70.6, c = 71.6, β = 100.9	a = 53.2, b = 69.7, c = 71.1, α = 88.4, β = 74.3, γ = 87.9
Resolution (Å)	49.83–1.75 (1.78–1.75)	48.36–1.75 (1.78–1.75)
R_{merge}	0.122 (0.511)	0.093 (0.327)
R_{pim}	0.049 (0.204)	0.067 (0.228)
Total reflections	338,363 (18,019)	341,066 (14,776)
Unique reflections	47,285 (2,505)	96,240 (4,648)
Mean $I/\sigma(I)$	10.4 (4.7)	7.1 (3.7)
$CC_{1/2}$	0.993 (0.942)	0.963 (0.872)
Completeness (%)	98.4 (95.4)	97.1 (93.6)
Multiplicity	7.2 (7.2)	3.5 (3.4)
Molecules/asymmetric unit	1	2
**Refinement**		
Resolution (Å)	49.88–1.75	47.67–1.75
No. of reflections (all/free)	47,231/2,396	96,224/4,810
R_{work}/R_{free}	0.174/0.215	0.156/0.193
Number of atoms	4,222	8,766
RMSD from ideal values		
Bond lengths (Å)	0.0102	0.0103
Bond angles (°)	1.65	1.70
Clashscore	2.71	2.03
Molprobity score	1.46	1.21
Ramachandran plot (%)		
Favored/allowed/outlier	96.34/2.85/0.81	96.92/2.29/0.80
PDB code	8K2F	8K2G

Values in parentheses are for the highest resolution shell.

Supplementary Table 3. Data collection and refinement statistics of the crystallography of NgaAt (Group 2)

Data set	NgaAt GalNAc-thiazoline	NgaAt GlcNAc-thiazoline
**Data collection**		
Beamline	BL32XU	BL32XU
Wavelength (Å)	1.0000	1.0000
Space group	$P2_12_12_1$	$P2_12_12_1$
Unit cell (Å/°)	a = 68.9, b = 134.0, c = 150.0	a = 69.3, b = 133.8, c = 150.7
Resolution (Å)	48.02–2.20 (2.25–2.20)	48.13–2.50 (2.58–2.50)
R_{merge}	0.226 (3.004)	0.270 (3.022)
R_{pim}	0.064 (0.873)	0.105 (1.158)
Total reflections	972,501 (60,305)	685,113 (64,372)
Unique reflections	71,289 (4,507)	49,358 (4,468)
Mean $I/\sigma(I)$	9.5 (1.9)	7.4 (1.4)
$CC_{1/2}$	0.997 (0.648)	0.994 (0.684)
Completeness (%)	100.0 (100.0)	100.0 (100.0)
Multiplicity	13.6 (13.4)	13.9 (14.4)
Molecules/asymmetric unit	2	2
**Refinement**		
Resolution (Å)	48.07–2.20	48.13–2.50
No. of reflections (all/free)	71,198/3,690	49,278/2,489
R_{work}/R_{free}	0.169/0.229	0.157/0.230
Number of atoms	9,285	9,217
RMSD from ideal values		
Bond lengths (Å)	0.0015	0.0149
Bond angles (°)	2.14	2.21
Clashscore	3.88	4.96
Molprobity score	1.73	2.02
Ramachandran plot (%)		
Favored/allowed/outlier	97.04/2.24/0.72	96.40/3.06/0.54
PDB code	8K2H	8K2I

Values in parentheses are for the highest resolution shell.

Supplementary Table 4. Data collection and refinement statistics of the crystallography of NgaDssm (Group 3)

Data set	NgaDssm apo	NgaDssm GalNAc-thiazoline
**Data collection** [a]		
Beamline	BL32XU	BL32XU
Wavelength (Å)	1.0000	1.0000
Space group	$P4_32_12$	$P4_32_12$
Unit cell (Å/°)	$a = b = 53.4$, $c = 427.4$	$a = b = 53.5$, $c = 428.3$
Resolution (Å)	47.75–1.75 (1.78–1.75)	47.88–1.75 (1.78–1.75)
R_{merge}	0.202 (1.872)	0.286 (3.351)
R_{pim}	0.043 (0.410)	0.061 (0.740)
Total reflections	1,489,931 (75,841)	1,515,006 (79,728)
Unique reflections	64,770 (3,512)	65,283 (3,545)
Mean $I/\sigma(I)$	10.6 (2.2)	8.7 (2.3)
$CC_{1/2}$	0.997 (0.688)	0.994 (0.629)
Completeness (%)	100.0 (100.0)	100.0 (100.0)
Multiplicity	23.0 (21.6)	23.2 (22.5)
Molecules/asymmetric unit	1	1
**Refinement**		
Resolution (Å)	47.80–1.76	47.93–1.75
No. of reflections (all/free)	63,071/3,242	65,066/3,313
R_{work}/R_{free}	0.180/0.213	0.176/0.206
Number of atoms	4,718	4,307
RMSD from ideal values		
Bond lengths (Å)	0.0154	0.0109
Bond angles (°)	2.01	1.65
Clashscore	3.38	2.90
Molprobity score	1.63	1.21
Ramachandran plot (%)		
Favored/allowed/outlier	95.60/3.25/1.15	95.60/3.63/0.76
PDB code	8K2J	8K2K

Values in parentheses are for the highest resolution shell.

Supplementary Table 5. Data collection and refinement statistics of the crystallography of NgaP2 (Group 4)

Data set	NgaP2 apo	NgaP2 GalNAc-thiazoline
**Data collection** [a]		
Beamline	BL32XU	BL32XU
Wavelength (Å)	1.0000	1.0000
Space group	*C*2	*C*2
Unit cell (Å/°)	*a* = 119.1, *b* = 60.4, *c* = 100.3, β = 120.1	*a* = 120.0, *b* = 61.6, *c* = 100.5, β = 121.4
Resolution (Å)	46.66–1.95 (2.00–1.95)	47.10–1.65 (1.68–1.65)
R_{merge}	0.137 (0.398)	0.139 (0.565)
R_{pim}	0.056 (0.172)	0.056 (0.229)
Total reflections	312,050 (21,086)	519,667 (25,463)
Unique reflections	45,106 (3,144)	74,805 (3,646)
Mean $I/\sigma(I)$	13.2 (10.3)	8.8 (3.6)
$CC_{1/2}$	0.986 (0.541)	0.987 (0.906)
Completeness (%)	99.9 (99.6)	99.3 (98.1)
Multiplicity	6.9 (6.7)	6.9 (7.0)
Molecules/asymmetric unit	1	1
**Refinement**		
Resolution (Å)	46.70–1.95	47.15–1.65
No. of reflections (all/free)	45,102/2,287	74,698/3,800
R_{work}/R_{free}	0.148/0.195	0.168/0.195
Number of atoms	4,900	4,971
RMSD from ideal values		
Bond lengths (Å)	0.0152	0.0120
Bond angles (°)	1.88	1.69
Clashscore	1.46	0.90
Molprobity score	1.37	0.88
Ramachandran plot (%)		
Favored/allowed/outlier	97.31/2.15/0.54	97.67/1.62/0.72
PDB code	8K2L	8K2M

Values in parentheses are for the highest resolution shell.

Supplementary Table 6. Data collection and refinement statistics of the crystallography of NgaLy (Group 4)

**Data set**	**NgaLy apo**
**Data collection** [a]	
Beamline	BL32XU
Wavelength (Å)	1.0000
Space group	*P*1
Unit cell (Å/°)	*a* = 82.9, *b* = 94.6, *c* = 116.2, α = 70.9, β = 73.8, γ = 72.4
Resolution (Å)	49.01–2.50 (2.54–2.50)
R_{merge}	0.099 (0.298)
R_{pim}	– (–)
Total reflections	215,567 (10,612)
Unique reflections	107,859 (5,311)
Mean $I/\sigma(I)$	7.1 (2.8)
$CC_{1/2}$	0.963 (0.672)
Completeness (%)	100.0 (100.0)
Multiplicity	2.0 (2.0)
Molecules/asymmetric unit	4
**Refinement**	
Resolution (Å)	48.79-2.50
No. of reflections (all/free)	107,859/5,287
$R_{\mathrm{work}}/R_{\mathrm{free}}$	0.183/0.241
Number of atoms	17,554
RMSD from ideal values	
Bond lengths (Å)	0.00151
Bond angles (°)	2.16
Clashscore	4.61
Molprobity score	2.06
Ramachandran plot (%)	
Favored/allowed/outlier	95.01/4.11/0.88
PDB code	8K2N

Values in parentheses are for the highest resolution shell.

Supplementary Table 7. Structural similarity between β-NGA groups compared using the root-mean-square distance (RMSD)

	RMSD (Å)						
	NgaCa	NgaAt	NgaDssm	NgaLy	NgaP2	CpGH123	BvGH123
NgaCa	0	3.36	5.57	8.06	4.51	7.04	6.71
NgaAt		0	3.60	11.16	11.51	13.86	9.09
NgaDssm			0	2.32	1.49	3.79	2.45
NgaLy				0	1.01	8.73	4.86
NgaP2					0	3.89	5.97
CpGH123						0	1.63
BvGH123							0

Supplementary Table 8. Amino acids conserved between β-NGA groups
Point mutation experiments were performed on the amino acids of NgaLy as shown in bold.

NgaCa	NgaAt	NgaDssm	NgaLy	NgaP2	BvGH123
stabilizer					
D287	D401	D353	D327	D359	D361
acid/base					
E288	E402	E354	E328	E360	E361
important residue					
D109	D134	D131	**D95**	D114	D132
W144	W157	W150	**W115**	W134	W152
Y363	Y495	Y422	**Y392**	Y424	Y427
G399	G530	G457	**G427**	G459	G460
W403	W534	W461	**W431**	W463	W464
Y434	Y564	Y494	**Y466**	Y498	Y491
R449	R580	R505	**R478**	R509	R499

Supplementary Table 9. The amounts of enzyme used in the point mutation assay.

	WT	D→E	D→N	E→D	E→Q
NgaDssm	0.1 µg	1 µg	1 µg	1 µg	0.1 µg
NgaCa	40 µg	400 µg	400 µg	400 µg	40 µg
NgaAt	5 µg	50 µg	50 µg	5 µg	5 µg
NgaLy	3 ng	300 ng	300 ng	300 ng	300 ng
NgaP2	45 ng	450 ng	450 ng	450 ng	450 ng

Supplementary Table 10. ^{1}H and ^{13}C NMR data of Galβ1-3GalNAcα/β.[a,b] The Galβ1-3GalNAcα/β used for the measurement were in the reaction mixture of Galβ1-3GalNAc-β-*p*NP with NgaDssm in D_2O [pH(D) 5.0] at 37°C. The sample contains about a 1.5:1 mixture of Galβ1-3GalNAcα/β after hydrolysis (Supplementary Figure 16)

Gal*p*β^1(α)[c]	^{1}H/ppm	*J*/Hz	^{13}C/ppm[d]	Gal*p*β^2(β)[c]	^{1}H/ppm	*J*/Hz	^{13}C/ppm[d]
1	4.49	d, 7.6	104.97	1	4.43	d, 8.0	104.56
2	3.51	dd, 10.0, 7.6	70.36	2	3.51	dd, 10.0, 8.0	70.36
3	3.61	dd, 10.0, 3.6	72.68	3	3.61	dd, 10.0, 3.6	72.68
4	3,90	d, 3.6	68.76	4	3,90	d, 3.6	68.76
5	3.63–3.68	m	74.93	5	3.63–3.68	m	74.93
6	3.70–3.79	m	60.96/61.19	6	3.70–3.79	m	60.96/61.19
	3.70–3.79	m	/61.66		3.70–3.79	m	/61.66
GalNAc*p*α	^{1}H/ppm	*J*/Hz	^{13}C/ppm	GalNAc*p*β	^{1}H/ppm	*J*/Hz	^{13}C/ppm
1	5.21	d, 3.6	91.37	1	4.69	d, 9.2	98.92
2	4.34	dd, 11.6, 3.6	51.23	2	3.98	dd, 11.2, 9.2	50.65
3	4.29	dd, 9.6, 3,2	71.17	3	3.84	dd, 11.2, 3.2	76.83
4	4.02	d, 3.2	68.97	4	4.17	d, 3.2	68.58
5	4.13	dd 6.4, 4,8	67.65	5	3.70–3.79	m	75.41
6	3.70–3.79	m	60.96/61.19	6	3.70–3.79	m	60.96/61.19
	3.70–3.79	m	/61.66		3.70–3.79	m	/61.66
Ac	2.01	s	22.20	Ac	2.01	s	22.20

a) 400 MHz at 21.2°C in D_2O presaturated and referenced using the peak of HOD at 4.80 and the native scale for ^{1}H and ^{13}C NMRs, respectively; b) The peaks of HEPES/DTT were appeared in ^{1}H NMR at δ 3.90, 3.75, 3.64, 3.23, 3.15, 2.98, 2.83–2.40 ppm; c)α and β in parentheses indicate the stereochemistry of the reducing GalNAc residue of the corresponding disaccharides; d) ^{13}C NMR data of the Galβ1-3GalNAcα/β were obtained using the HMQC spectrum (Supplementary Figures 7 C and D).

Supplementary Table 11. ^{1}H and ^{13}C NMR data of Galβ1-3GalNAc-β-*p*NP (Supplementary Figure 17)[a)]

D-Gal*p*β	^{1}H/ppm	*J*/Hz	^{13}C/ppm	Key HMBC
1	4.48	d, 7.2	104.94	→ Irr. (79.3, C3$^{D\text{-}GalNAc}$)
2	3.54	dd, 9.2, 7.2	70.62	
3	3.62	dd, 9.2, 2.0	72.51	
4	3,91	br s	68.63	
5	3.64–3.69	m	75.10	
6	3.93	dd 12.4, 7.2	61.07	
	3.78	dd 12.4, 3.6		
β-D-GalNAc*p*	^{1}H/ppm	*J*/Hz	^{13}C/ppm	HMBC
1	5.30	d, 8.8	98.92	
2	4.34	dd, 9.6, 8.8	51.04	
3	4.03	dd, 9.6, 1.6	79.34	→ Irr. (104.94, C1$^{D\text{-}Galp\beta}$)
4	4.28	br s	67.84	
5	3.92–3.96	m	75.39	
6	3.84	dd 12.4, 6.4	60.72	
	3.79	dd 12.4, 5.6		
Ac	1.99	s	22.20	
	-	-	175.17	
*p*NP	8.23–8.25	m	126.14	
	7.18–7.21	m	116.58	
	-	-	161.84, 142.66	

a) 400 MHz at 21.2°C in D_2O referenced using the peak of HOD at 4.80 and the native scale for ^{1}H and ^{13}C NMRs, respectively.
