# Supplementary material for: Genetic and functional diversity of β-N-acetylgalactosamine-targeting glycosidases expanded by deep-sea metagenome analysis
Source: Nat Commun. 2024 May 10;15:3543. doi: 10.1038/s41467-024-47653-2 (PMC11087588; doi:10.1038/s41467-024-47653-2)
Supplement: Supplementary file 3 — Description of Additional Supplementary Files [file 41467_2024_47653_MOESM3_ESM.pdf]

Description of Additional Supplementary Files

File Name: Supplementary Data 1
Description: Candidate β-NGA sequences retrieved from deep-sea sediment metagenomes.

File Name: Supplementary Data 2
Description: Sequences used for the phylogenetic tree of β-NGA candidates in Fig.1c.

File Name: Supplementary Data 3
Description: Select amino acid sequences for expression analysis.

File Name: Supplementary Data 4
Description: Neighborhood genes of β-NGAs.

File Name: Supplementary Data 5
Description: Predicted functional partners proteins from the STRING database.

File Name: Supplementary Data 6
Description: Cloning and mutation primers for PCR.

File Name: Supplementary Data 7
Description: Crystallization solutions of the β-NGAs.
